# Biodiversity Can Help Prevent Malaria Outbreaks in Tropical Forests

**DOI:** 10.1371/journal.pntd.0002139

**Published:** 2013-03-21

**Authors:** Gabriel Zorello Laporta, Paulo Inácio Knegt Lopez de Prado, Roberto André Kraenkel, Renato Mendes Coutinho, Maria Anice Mureb Sallum

**Affiliations:** 1 Departamento de Epidemiologia, Faculdade de Saúde Pública, Universidade de São Paulo, São Paulo, São Paulo, Brazil; 2 Departamento de Ecologia, Instituto de Biociências, Universidade de São Paulo, São Paulo, São Paulo, Brazil; 3 Instituto de Física Teórica, Universidade Estadual Paulista Júlio de Mesquita Filho, São Paulo, São Paulo, Brazil; University of Notre Dame, United States of America

## Abstract

**Background:**

*Plasmodium vivax* is a widely distributed, neglected parasite that can cause malaria and death in tropical areas. It is associated with an estimated 80–300 million cases of malaria worldwide. Brazilian tropical rain forests encompass host- and vector-rich communities, in which two hypothetical mechanisms could play a role in the dynamics of malaria transmission. The first mechanism is the dilution effect caused by presence of wild warm-blooded animals, which can act as dead-end hosts to *Plasmodium* parasites. The second is diffuse mosquito vector competition, in which vector and non-vector mosquito species compete for blood feeding upon a defensive host. Considering that the World Health Organization Malaria Eradication Research Agenda calls for novel strategies to eliminate malaria transmission locally, we used mathematical modeling to assess those two mechanisms in a pristine tropical rain forest, where the primary vector is present but malaria is absent.

**Methodology/Principal Findings:**

The Ross–Macdonald model and a biodiversity-oriented model were parameterized using newly collected data and data from the literature. The basic reproduction number (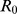) estimated employing Ross–Macdonald model indicated that malaria cases occur in the study location. However, no malaria cases have been reported since 1980. In contrast, the biodiversity-oriented model corroborated the absence of malaria transmission. In addition, the diffuse competition mechanism was negatively correlated with the risk of malaria transmission, which suggests a protective effect provided by the forest ecosystem. There is a non-linear, unimodal correlation between the mechanism of dead-end transmission of parasites and the risk of malaria transmission, suggesting a protective effect only under certain circumstances (e.g., a high abundance of wild warm-blooded animals).

**Conclusions/Significance:**

To achieve biological conservation and to eliminate *Plasmodium* parasites in human populations, the World Health Organization Malaria Eradication Research Agenda should take biodiversity issues into consideration.

## Author Summary

*Plasmodium vivax* malaria is a neglected infectious disease that can cause severe symptoms and death in tropical regions. It is associated with an estimated 80–300 million cases of malaria worldwide. Brazilian tropical rain forests are home to a rich community of animals that can participate in the dynamics of malaria transmission. In this study, we used real data and computer simulation to study two aspects of biodiversity (an increase in the abundance of wild warm-blooded animals; and an increase in the abundance of non-malarial mosquitoes) and the effects they have on malaria outbreaks. We found that both aspects can help prevent malaria outbreaks in tropical forests. We also found that a decrease in the abundance of wild warm-blooded animals can increase the population of malarial mosquitoes and thus increase the chances of malaria outbreaks. Forest conservation and malaria control are not incompatible and thus biodiversity issues should be included in the World Health Organization Malaria Eradication Research Agenda in order to achieve the desirable goals of biological conservation and maintenance of low malaria transmission.

## Introduction

The dynamics of malaria transmission involve a tritrophic interaction among vector mosquitoes (*Anopheles* species), protozoan parasites (*Plasmodium* species), and vertebrate hosts. Malaria is endemic in tropical and subtropical regions [1]–[3]. The Global Malaria Eradication Program adopted in the 1950s has failed to meet expectations for malaria control in tropical and subtropical countries. One of the causes of that failure was the lack of an in-depth knowledge of the ecology of malaria-parasite transmission [4]. In addition, *Plasmodium vivax* malaria has been neglected as a chronic disease [5].

The prevalence of malaria remains high, especially in Africa, the Americas, Asia, and the western Pacific. In those regions collectively, the prevalence was 2% in 2011, most cases occurring in children [6]. Recently, Murray et al. suggested that, although malaria mortality rates have remained stable worldwide, the World Health Organization underestimated malaria mortality for the last two decades, purporting that the number of deaths from malaria among adults in Africa, as well as among adults and children outside of Africa, was substantially higher than that reported [7]. Because of the suffering caused for malaria to humans mainly in developing countries, elimination of this disease is a challenge for the Malaria Eradication Research Agenda [8]. According to the World Health Organization agenda for vector control, there is an urgent need to identify key knowledge gaps in vector ecology and biology [9]. Such knowledge will be important to define strategies for mosquito control, as well as to reduce the number of infective bites and the basic reproduction number [8]. The basic reproduction number (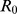) is the expected number of secondary cases arising from a single case in a given susceptible population and is used as a measure of malaria-parasite transmission, as well as of the impact of control programs.

In the forested areas of the biogeographical subregion known as the Serra do Mar (mountain range), within the Atlantic Forest of southeastern Brazil [10], where the levels of insect and vertebrate richness are high [11], [12], malaria is hypoendemic [13]–[16], and the primary malaria parasite being *Plasmodium vivax* [17]. In the Atlantic forest, species of the *Anopheles* subgenus *Kerteszia* are the primary malaria vectors. The majority of *Kerteszia* species use bromeliad-phytotelmata as larval habitats [18], and it has been suggested that *Kerteszia* spp. participate in the dynamics of malaria-parasite transmission in Trinidad [19] and along the Atlantic coast of Brazil [20], [21]. Between 1944 and 1951, there were malaria epidemics in the southern Atlantic Forest within the states of Santa Catarina and Paraná, the overall incidence for the period being 5% [21]. Such epidemics mainly ceased because of deliberate deforestation that eliminated 3,800 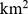 of native forest [22], removing bromeliads and reducing the number of resting sites for adult mosquitoes within the forest [21].

Although malaria epidemics are currently uncommon, temporal and spatial clustering of cases can occur in the Atlantic Forest. One low-incidence outbreak occurred among outdoor workers in the forested highlands of the state of Espírito Santo between 2001 and 2004 [23]. In 2006, another epidemic occurred in the southern periphery of the city of São Paulo, where residents of the Marsilac district invaded the Serra do Mar Natural Forest Reserve to construct houses [24]. Given the presence of *Anopheles* vector species, as well as that of infected and susceptible hosts, together with the circulation of *Plasmodium*, it is hypothesized that ecological interactions among *Anopheles* (*Kerteszia*) *cruzii*, *Plasmodium* species, and the local biodiversity are modulating malaria transmission in the Serra do Mar.

Forested areas offer a diverse range of habitats for mosquito species [25]. Consequently, high levels of mosquito species richness and abundance are expected [11]. This scenario can decrease the number of infective bites, because multiple vector and non-vector mosquito species would try to feed on a defensive host [26], [27], decreasing the chances of successful bites by the vector population. In addition, malaria parasite transmission could be affected by an abundance of non-competent hosts that would prevent mosquitoes from transmitting *Plasmodium* parasites to humans [28]. This would represent a dilution effect of wild warm-blooded animals, which act as dead-end hosts [29]. Therefore, diffuse mosquito vector competition and dead-end transmission of parasites are mechanisms in the dynamics of malaria transmission in tropical forests that, consequently, can alter the chances of malaria emergence.

The insight that ecological mechanisms can influence the dynamics of malaria parasite transmission supports arguments against human occupation of protected natural areas. Current theory says that biodiversity can have an impact on the emergence and transmission of infectious diseases, which is a new focus of conservation studies [30]. Some authors have shown that when biodiversity declines, there is an increased risk of humans contracting schistosomiasis [31], West Nile fever [32], hantavirus infection [33], or Lyme disease [34]. Similarly, diseases that affect coral reefs become more widespread when biodiversity is reduced by human activities [35]. The relationship between a decline of biodiversity and an elevated risk of vector-borne disease might be attributable to changes in the abundance of hosts and vectors or to modified host, vector, or parasite behavior [30]. In the Brazilian Amazon, gradual and continuous changes in the natural ecosystems can create ecological conditions that favor a rapid increase in abundance of *Anopheles darlingi*, which have been associated with an increased risk of malaria. Sawyer and Sawyer coined the term “frontier malaria” to define the dynamics of malaria transmission in recently deforested areas of the Amazon Forest [36]. In those areas, malaria transmission decreases when the natural ecosystem is highly modified, to the point that the maintenance of vector species and *Plasmodium* circulation are not ecologically supported [36], [37]. Therefore, malaria in Amazon and in the Atlantic Forest are both associated with biodiversity, because the larval habitats of *An. darlingi*, the primary vector in Amazon Region, and *An. cruzii*, the primary vector in Atlantic Forest, depend on the presence of forested areas.

Historically, tropical regions have been considered economically underdeveloped hotspots of biodiversity [38]. However, Brazil is now becoming an emerging global economy, which suggests that its forest cover, along with its biodiversity, will decline rapidly [39]. If this occurs, frontier malaria will be eliminated, which would increase the risk of rural and urban malaria alike, because *Anopheles marajoara* could become a vector of *Plasmodium* parasites [40], [41]. Given that malaria cannot be completely eliminated and that there is an urgent need for conservation/restoration of tropical biodiversity, it is important to understand interactions between the dynamics of malaria transmission and the diversity of vertebrates and mosquitoes. Here, we employed a mathematical model to develop a theoretical framework that might explain how biodiversity can modulate malaria epidemics in a tropical rain forest. Our case study site is a protected area within the Atlantic Forest, inhabited by indigenous peoples and fishermen, where *An. cruzii* is present but no malaria cases have been reported in the last 30 years. Our objectives were to propose a novel mathematical model for malaria transmission with explicit mechanisms of diffuse mosquito vector competition and dead-end transmission of parasites, applying this model to this case study site, as well as assessing how diffuse competition among mosquito vectors and dead-end transmission affect malaria epidemics.

## Materials and Methods

### Study area

**Figure 1:**
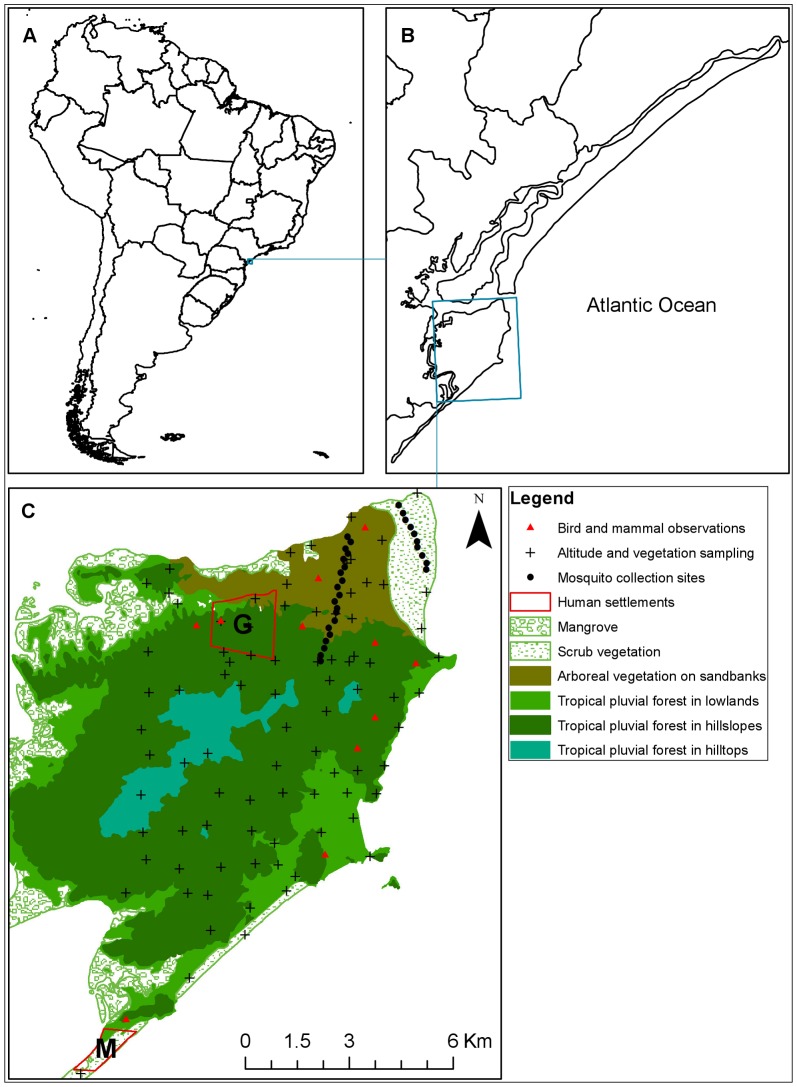
Study area. A: South America and Brazilian States; B: The Iguape-Cananéia-Paranaguá estuarine lagoon region, southeastern coast of Brazil; and C: Parque Estadual da Ilha do Cardoso. G, The Guarani Mbya village; and M, Marujá. Source: Bird and mammal observations [45]; Altitude and vegetation sampling [46] (Figure S6).

The Iguape-Cananéia-Paranaguá estuarine lagoon region is a coastal plain area of approximately 22,600-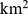, situated on the southeastern coast of Brazil (Figure 1A), between the Ribeira de Iguape River and the Atlantic Ocean (Figure 1B). Three great islands stretch along the coast a hundred kilometers from northeast to southwest, namely Comprida, Cananéia, and Cardoso [42]. Cardoso island, hereafter referred to as Parque Estadual da Ilha do Cardoso (PEIC), is a São Paulo State Park within the Atlantic Forest [43].

The PEIC is separated from the mainland by the Ararapira Channel, a body of water that is as narrow as 30 m wide at places. Therefore, even large animals, such as muriqui (*Brachyteles*) can cross [44]. Common wild warm-blooded animals include medium to large birds, such as quail (*Odontophorus capueira*), toucans (*Ramphastos* species), guans (*Penelope* species and *Pipile jacutinga*), tinamous (*Tinamus solicarius*), and mammals, such as howler monkeys (*Alouatta* species), agoutis (*Dasyprocta leporina*), and squirrels (*Sciurus ingrami*), as described by Bernardo [45]. Vegetation types form a successional gradient from sand dunes at the shore to higher and ancient terrains inland. Along the coastal plain, there is sand dune vegetation, scrubland, and low forests with sandy soil (arboreal restinga, or shoal vegetation). As can be seen in Figure 1C, tropical pluvial forest vegetation types are found on hillsides and hilltops [46].

Descendents of European colonists previously occupied what is now the PEIC, and the major local activities were fishing and family farming [47]. The population density is currently approximately 3.3 people/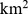, which has no relevant impact on local biodiversity. However, tourism has become one of the main sources of income, and thousands of tourists arrive every summer in the fishing village of Marujá, to the south (Figure 1C). In addition, the indigenous Guarani Mbya tribe has been settled in the northwestern part of the PEIC since 1992, having the right to engage in subsistence hunting and logging in the forest [48], [49].

*Anopheles cruzii*, i.e., a primary malaria vector in the Atlantic Forest, is present in PEIC. Although no malaria cases have been reported in the last 30 years in PEIC, *P. vivax* is circulating in the immediate surrounding region [13], [15], [17] (Figure S1). Therefore, it is plausible to assume that introduction of *Plasmodium* species can occur in the region because of the thousands of tourists that visit the PEIC during the summer, including those traveling from endemic areas.

### Model of malaria transmission

The mathematical model proposed herein represents parasite transmission among four compartments that play specific roles in the dynamics of malaria transmission in the Atlantic Forest.

The first compartment is susceptible human populations in the Guarani and Marujá settlements, which were set at constant sizes. Considering that few humans are allowed to live in the PEIC, the size of the human population size remains approximately constant. However, malaria could emerge in the area because of its location in the Serra do Mar, where low-level endemic parasite transmission occurs [13], [15]. In addition, it is also plausible to assume that introduction of *Plasmodium* species can occur in the region because of the 15,000 tourists that visit the PEIC during the summer [48], including those traveling from endemic areas. Consequently, the human population from PEIC can be exposed to malaria parasites.

The human risk of *Plasmodium* infection in PEIC depends on the *An. cruzii* biting rate [50] and the probability of an infective bite [51], [52]. Therefore, a proportion of susceptible humans are included in the second compartment, infected humans. For this simple vector-human malaria parasite transmission, however, ecological interactions can be added in order to create a more realistic scenario of malaria transmission. Other mosquito species and a diversified vertebrate community [12], [45] (Table S1 and Table S2) are intermixed, competing for food and spatial resources with mosquito vectors and humans. Therefore, human malaria transmission may become difficult because of the increased abundance of non-vector mosquitoes and of vertebrate animals. Infectious humans can recover, becoming again susceptible to infection with malaria parasites. This assumption is based on the lack of human cross-immunity against *Plasmodium* species that circulate in the Atlantic Forest.

Until infectious humans are cured of the infection, they can infect *An. cruzii* [50] with a given probability of infection [51], [52]. In the third compartment, *An. cruzii* population was assumed to be in equilibrium, meaning that its mean mortality rate [50] was assumed to be lower than its hatch rate because of its high abundance in forested areas of the Atlantic Forest [14], [21]. The *An. cruzii* hatch rate is dependent on successful bites on animals and humans. Biting success was expressed by the mean period of free biting until the occurrence of host defensive behavior (Figure S2). Susceptible *An. cruzii* in the third compartment may be infected by means of biting infectious humans. Non-vector mosquito species decrease the risk of human-vector contact, whereas increased numbers of animals and humans can increase vector abundance. In the forth compartment, *An. cruzii* is now infected by *Plasmodium* and can transmit it back to humans.

### Mathematical model of malaria transmission

To calculate the risk of malaria parasite transmission, we used our model and the Ross–Macdonald model for the two most populated human settlements, the Guarani village and Marujá (Figure 1C). With the Ross–Macdonald model, it was assumed that *An. cruzii* abundance was constant; that is, there were no ecological interactions [53]. Therefore, the model was mathematically expressed as follows [54]:
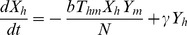
(1)

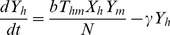
(2)

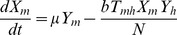
(3)

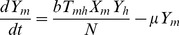
(4)where 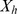 = susceptible humans; 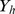 = infected humans; 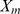 = susceptible mosquitoes; 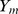 = infected mosquitoes; 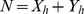; 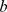 = biting rate; 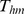 = transmission probability from a biting infected mosquito to a human; 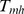 = transmission probability from a infected human to a biting mosquito; 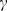 = recovery rate by humans; and 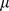 = mortality rate of vector mosquitoes.

To add ecological interactions, such as diffuse mosquito vector competition and dead-end transmission of parasites, we based our model on the Ross–Macdonald model [54]. First, we transformed the transmission factor in the Ross–Macdonald model (i.e., 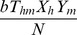) in another in which malaria-parasite transmission can be blocked by defensive hosts and non-vectors (i.e., diffuse mosquito vector competition) and non-hosts (i.e., dead-end transmission of parasites):
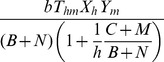
(5)where 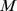 = abundance of *An. cruzii* females, 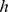 = biting tolerance exhibited by vertebrate animal and human hosts; 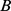 = abundance of wild warm-blooded animals; and 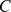 = abundance of non-vector mosquito females. Second, we considered that population dynamics of *An. cruzii* females was present:
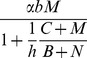
(6)where the rationale is that a successful bite is needed in order to start a new mosquito generation in the larval habitat in which density-dependent mechanisms can occur. It includes the following factors: female adult recruitment in the larval habitat (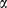) and female adult activity plus interactions with hosts and non-vectors 
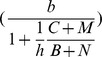
. The 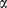 parameter is a measure of recruitment of adult female emergence and it can be fitted with abundance data collected in the field. Predation and competition which are strong components of mosquito populations in the larval habitat are implicitly considered in this 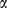 parameter. Abundance of *An. cruzii* adult females was assumed to be associated with life conditions and interactions in the larval habitat to estimate 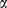. This association means that high abundance of adult females is expected when optimum physical, chemical and biological conditions are present in the larval habitat, or *vice-versa*. Abundance data was obtained with automatic CDC-CO_2_ traps which collect a sample of species (i.e., host-seeking females) in mosquito community. This automatic trap do not mimic host defensive behavior and diffuse competition which were thus made explicit in the population dynamics of *An. cruzii* females 
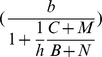
. Third, we estimated 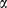 considering that the abundance of *An. cruzii* is in equilibrium (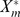) and utilizing the following equation:
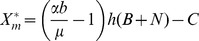
(7)where the underlying assumption is that competition affects both *An. cruzii* and non-vector mosquito species, leading to a situation in which vector and non-vector mosquito species can coexist and *An. cruzii* is therefore not excluded by competition.

Therefore, our final model for abundance of individuals in each compartment is as follows:
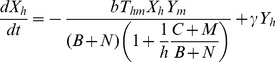
(8)

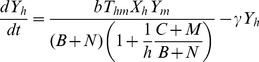
(9)

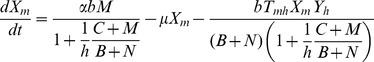
(10)

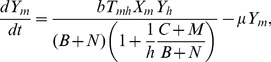
(11)where 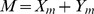.

The Ross–Macdonald model is a special case of our model if we consider that wild warm-blooded animals are either absent or do not interact with *An. cruzii* (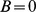); non-vector mosquito species are absent or do not interact with *An. cruzii* (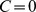); humans do not react to mosquito bites (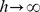); and *An. cruzii* abundance is constant (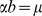). Analyses performed using our model had parameter values as inputs in order to calculate the basic 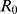 as an output (Text S1, Text S2). The goal of the analyses was to examine the relationship between malaria transmission dynamics (synthesized by 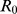 estimate) and hypotheses regarding ecological interactions (formalized into a model structure).

Finally, an intermediate model, in which both *An. cruzii* and non-vector mosquito species have constant populations, was elaborated (Text S3).

## Results

On the basis of collected data and data from the literature, the models described in the Materials and Methods section were completely parameterized for the case study site (Text S1). Empirical values were unavailable only for the host tolerance (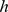) parameter, which was set to a range of permissible values and submitted to a sensitivity analysis (Text S1).

The indigenous settlement in the study area (a village occupied by members of the Guarani Mbya tribe) is inhabited by 150 natives in a 2.8-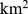 area, whereas 165 people (fishermen and their families) live in Marujá in a 0.8-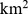 area (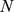 parameter; Figure S3). As can be seen in Table 1 and in the Figure S4, Figure S5 and Table S1, the estimated abundance of wild birds and mammals (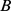 parameter) was 172 in The Guarani Mbya village and 47 in Marujá. The estimated abundance of mosquitoes in The Guarani Mbya village and Marujá, respectively, was 1,514 and 300 for the malaria vector *An. cruzii* (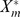 parameter), compared with 14,101 and 3,640 for non-vectors (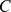 parameter), which were supported by Figure S6, Figure S7 and Table S3.

**Table 1:** Parameters, descriptions, estimates and references of the mathematical model of malaria transmission.

Parameter simbology	Description	Estimates	Reference
Human population size ( 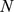 )	Total number of inhabitants in The Guarani Mbya village and Marujá	150 and 165, respectively	Text S1
Abundance of wild warm-blooded animals ( 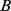 )	Estimates of abundance of avian and mammalian species in The Guarani Mbya village and Marujá	172 and 47, respectively	[45], Text S1
Abundance of non-vector mosquito 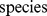 a ( 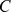 )	Estimates of abundance of non-vector mosquito species in The Guarani Mbya village and Marujá	14,101 and 3,640, respectively	Text S1
Abundance of 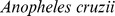 b ( 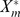 )	Estimates of abundance of *An. cruzii* in The Guarani Mbya village and Marujá	1,514 and 300, respectively	Text S1
*Anopheles cruzii* biting rate ( 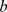 )	Biting rate of each *An. cruzii* female upon a given host per day	0.50	[50], Text S1
*Anopheles cruzii* mortality rate ( 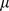 )	Mortality rate of *An. cruzii* female population per day	0.80	[50], Text S1
*Anopheles cruzii* convertion rate ( 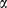 )	Convertion rate of a successful bite upon a host to the number of emerging females in The Guarani Mbya village and Marujá	5.5 and 3.1, respectively	Text S1
Probability of *Plasmodium* transmission from *Anopheles cruzii* to humans ( 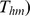	Probability of *Plasmodium* transmission from *An. cruzii* to humans in low-endemicity malaria transmission dynamics	0.022	[51], [52]
Probability of *Plasmodium* transmission from humans to *Anopheles cruzii* ( 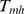 )	Probability of *Plasmodium* transmission from humans to *Anopheles cruzii* in low-endemicity malaria transmission dynamics	0.24	[51], [52]
Human recovery rate ( 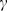 )	Daily human recovery rate, which can be understood as the average duration of the infectious period	0.0035 (286 days)	[51], [52] and Text S1
Host tolerance ( 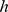 )	Number of bites per day before a host starts a defensive behavior divided by *An. cruzii* biting rate (0.5)	20, i.e., host defensive behavior occur after the 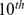 bite in a given day	Text S1

a : *Aedes serratus*, *Limatus durhami*, *Runchomyia reversa* and *Wyeomyia quasilongirostris*.

b : *Anopheles cruzii* is the primary vector of malaria *P. vivax* and *P. malariae* parasites [13].

Data in the literature, from laboratory and field experiments, show that the estimated maximum *An. cruzii* biting rate (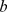 parameter) is 0.5 bites/day (Table 1). In the laboratory, we found the estimated gonotrophic cycle to be four days, and our field experiments indicated gonotrophic discordance in natural populations (Table 1). In our laboratory experiments, *An. cruzii* mortality (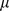 parameter) was estimated to be 0.80/day (Table 1). Employing the previously mentioned data from the literature, we estimated the 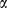 parameter, which indicates how many new adults will be generated from a single successful bite of *An. cruzii*, to be 5.5 in The Guarani Mbya village and 3.1 in Marujá (Table 1).

The estimated probabilities of *Plasmodium* species transmission and the human recovery rate correspond to the dynamics of transmission in a low-endemicity area (Table 1). Mosquitoes transmit malaria parasites to humans with a probability of 0.022 (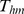 parameter), humans infect mosquitoes with a probability of 0.24 (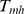 parameter), and the human recovery rate (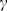 parameter) is 0.0035/day, which means that average duration of the infectious period is 286 days (Table 1).

The 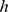 parameter was derived from two other values: the number of bites/day before a host starts a defensive action (assumed herein to be 10 bites/day), divided by the maximum biting rate (0.5 bites/day for *An. cruzii*). Another way to interpret the 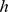 parameter is that host defensive behavior will be stronger when the average biting rate is greater than 10 bites/day (Table 1). On the basis of our field work experience in tropical forests (mainly the Atlantic Forest), we can state that neither humans nor animals can stand mosquito biting rates greater than approximately 10 bites/day before they begin to exhibit defensive behavior (e.g., shaking body parts or, in the case of humans, waving hands and swatting). Therefore, 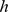 was set at 20. Mosquito vector diffuse competition and dead-end parasite transmission patterns were assessed for 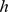 values within 20 and 30 (e.g., 21, 25, and 29) in the sensitivity analysis (Figure S8, Figure S9 and Figure S10). As a result, no qualitative changes could be made in the initial interpretations (see the following paragraphs).

The basic 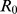 (Text S2), as calculated by the Ross–Macdonald model is as follows:
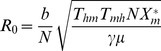
(12)whereas the basic 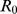 predicted by our model is the following:
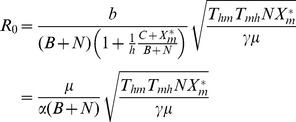
(13)where no malaria cases are to be reported when 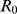 is 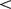
1 and if it exceeds 1 (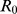 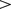
1) then the disease can invade and there should be an epidemic episode.

**Figure 2:**
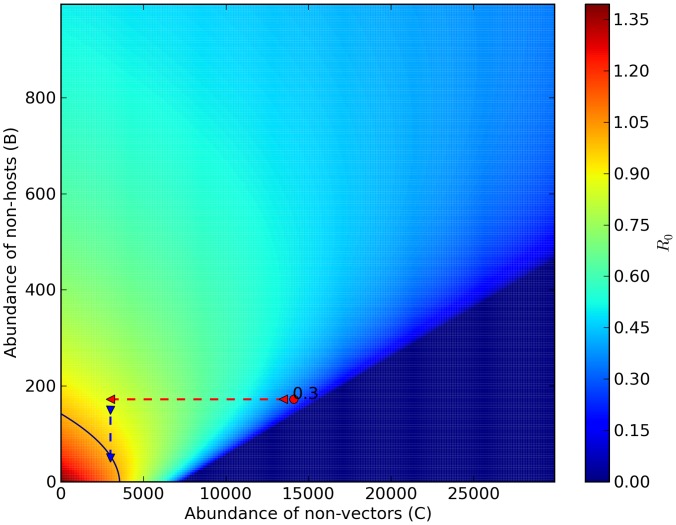
Predicting hypothetical scenarios I: dilution effect and diffuse mosquito vector competition in The Guarani Mbya village. Increase in abundance of non-vector mosquito species and in abundance of wild warm-blooded animals is correlated with decrease in the risk of malaria-parasite transmission. Reduction in abundance of wild warm-blooded animals (blue dashed arrow) and in abundance of non-vector mosquito species (red dashed arrow) can exceed the critical threshold level (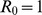). The red circle is 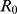 estimate of our model (0.3; eq. 13). The black isoline represents malaria transmission threshold (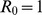). Color legend shows a range of 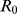 values from 0.00 to 1.40.

**Figure 3:**
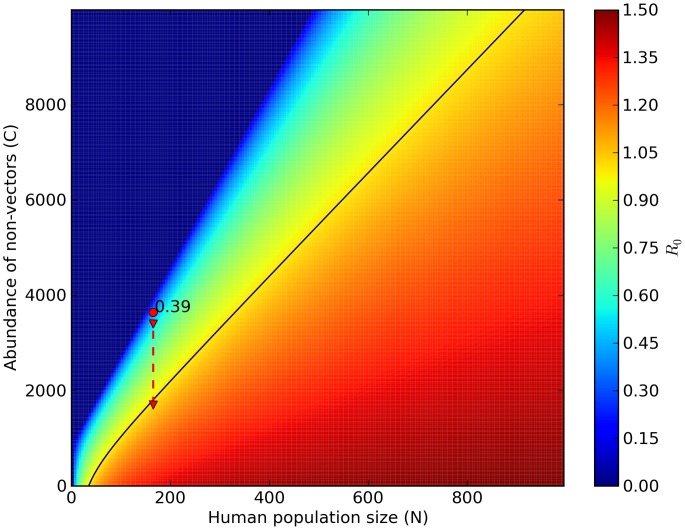
Predicting hypothetical scenarios II: diffuse mosquito vector competition in Marujá. Increase in abundance of non-vector mosquito species is linearly correlated with decrease in the risk of malaria-parasite transmission. Reduction in abundance of non-vector mosquito species (red dashed arrow) can exceed the critical threshold level (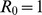). The red circle is 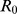 estimate of our model (0.39; eq. 13). The black isoline represents malaria transmission threshold (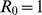). Color legend shows a range of 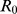 values from 0.00 to 1.50.

**Figure 4:**
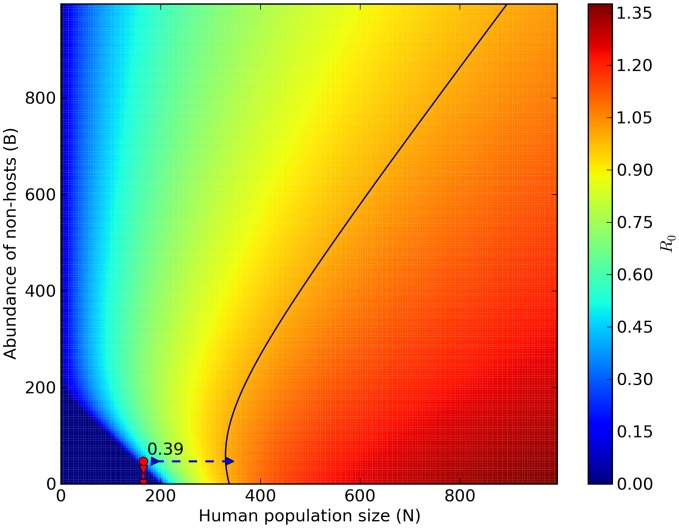
Predicting hypothetical scenarios III: dilution effect in Marujá. Increase in abundance of wild warm-blooded animals is non-linearly correlated with decrease in the risk of malaria-parasite transmission. Reduction in abundance of wild warm-blooded animals (red dashed arrow) does not exceed the critical threshold level (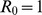). However, increase in human population size (blue dashed arrow) can exceed the critical threshold level (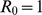). The red circle is 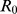 estimate of our model (0.39; eq. 13). The black isoline represents malaria transmission threshold (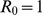). Color legend shows a range of 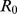 values from 0.00 to 1.40.

If we employ the 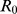 estimate relative to the Ross–Macdonald model (eq. 12), The Guarani Mbya village would have an 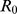 of 2.18 and Marujá would have an 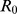 of 0.93. Using the model employed in the present study (eq. 13), we found the 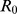 to be 0.30 for The Guarani Mbya village and 0.39 for Marujá. If the abundances of non-vector mosquito and non-host vertebrate species were reduced by approximately 80% and 70%, respectively, the critical threshold level (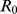 = 1) would be exceeded in The Guarani Mbya village (Figure 2). Similarly, a 50% reduction in the abundance of non-vector mosquito species could cause malaria invasion (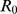 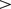
1) in Marujá (Figure 3). However, epidemics would not occur in Marujá even if there were local extinction of all non-host vertebrate species but would occur if the human population increased by approximately 50% (Figure 4).

Additionally, new simulations utilizing the intermediate model (Text S3) supported the previous statements (Figure S11) and showed that the original results are robust.

## Discussion

Although scarce malaria cases are reported annually in the surrounding area [13], [15], [17], no malaria cases have been reported in the last 30 years, being assumed that *Plasmodium* transmission does not occur in Parque Estadual da Ilha do Cardoso. The estimated 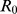 provided by Ross–Macdonald model (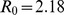) suggests that malaria parasite transmission may be occurring in that region. Contrasting, based on the model proposed herein, estimated 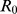 for The Guarani Mbya village was 0.30, which is in accordance with assumption of no malarial transmission. This model simulations (i.e., predicting hypothetical scenarios) were performed in order to show what would happen with 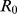 estimate value if: 1) Non-vector populations increase (i.e., effect of diffuse mosquito vector competition), 2) Non-host populations increase (i.e., effect of dead-end transmission of parasites), and 3) Human population increases (effect of over-encroachment of human populations). Simulation results provide support for biodiversity preventing the circulation of *P. vivax* in human settlements embedded in natural ecosystems. The absence of malaria cases can be explained by the diffuse mosquito vector competition and dead-end transmission of parasites provided by high abundances of mosquitoes and vertebrates. Greater abundances of mosquitoes and vertebrates can be correlated with higher levels of biodiversity, which increase ecosystem's functional redundancy, thus decreasing the chances of malaria occurrence, which is in keeping with the insurance hypothesis [55]. According to this hypothesis, an insurance effect is the ability of an ecosystem to buffer perturbations (e.g., *P. vivax* circulation), as well as the ability of the species in the community to respond differentially to perturbations (e.g., diffuse mosquito vector competition and dead-end transmission of parasites). Therefore, these mechanisms that hinder malaria parasite transmission are services provided by the forest ecosystems.

In view of the results of simulations conducted using the models applied in the present study (Figure 2,3), we suggest that increasing non-vector mosquito abundance can reduce the number of *An. cruzii* bites, decreasing malaria parasite transmission in the Atlantic Forest. A new law of mosquito-host relationship 
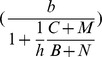
 is proposed here and it is supported by the following evidences: 1) there must be an intense selection pressure on hosts to exhibit defensive behavior against biting insects [56], [57], and 2) contacts between mosquito species and specific hosts in a community may be influenced more by the presence/absence of hosts than by innate mosquito choices [58]. This law can be defined as a community of defensive hosts in which the access to their blood is a limiting resource, providing competition among opportunistic blood-feeder mosquito species. The total abundance of non-vector and not-infected vector mosquito species can have a negative impact on malaria parasite transmission because of apparent competition mediated by host defensive behavior. The effect of apparent competition is a functional response that may be associated with host tolerance to mosquito bites. When the host tolerance threshold is reached, mosquito bites are avoided by defensive responses from the host. The presence of non-vector and not-infected vector mosquitoes seems to propitiate a larger number of unsuccessful bites, with few *Plasmodium*-infective bites. The vector competition effect could also occur within species. For example, when there is more larval habitat available (during the wet season), hatch rates increase, making the proportion of nulliparous females larger than that of parous females. Host defensive behavior was observed for blacklegged ticks that are killed when feeding on the blood of opossums and squirrels [34]. Consequently, diffuse competition is a protective mechanism against infective bites and should therefore be considered a major factor in studies related to the dynamics of malaria transmission. In considering that *Plasmodium* species infection can affect the feeding behavior of anthropophilic mosquitoes [59], it would be important to understand how the mechanism of diffuse competition can be applied to malaria control strategies in endemic tropical regions.

The abundance of wild warm-blooded animals can decrease the transmission of *Plasmodium* species. Such animals can act as dead-end hosts, diminishing the chances of infective bites in humans, which can be used as an indirect method of malaria control. This might represent a dilution effect mechanism present in natural ecosystems that have a high abundance of warm-blooded animal species. Dilution effect mechanisms [29] were observed by Swadle and Calos [32], Johnson et al. [31], and Suzán et al. [33] for West Nile fever, schistosomiasis, and hantavirus infections, respectively. However, a low- to medium-level abundance of dead-end hosts can create a neutral situation in which the dilution effect is either unimportant [60] or harmful [28]. Using a computer simulation, Allan Saul showed that the dilution effect (zooprophylaxis) can be harmful when a small number of dead-end hosts potentiate malaria parasite transmission by providing blood-feeding opportunities to vectors [28]. Our model predicts that few wild warm-blooded animals can serve as blood sources for mosquito species, increasing the vector population and *Plasmodium* species dissemination. This can be seen in the non-linear unimodal relationship between the abundance of non-hosts and the critical threshold level (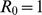), as depicted in Figure 4. This finding is supported by the work of Randolph and Dobson, who stated that the dilution effect applies only to species-rich host communities in which there is variable reservoir competence [61]. In addition, hunting activities that are allowed for traditional human communities in natural protected conservation units can reduce vertebrate abundance, whereas it increases the density of vegetation and the abundance of invertebrates, resulting in the so-called “empty forest” effect [62] and increasing the chances of malaria parasite transmission.

Having the present model as a starting point, two new avenues can be pursued for studying dynamics of malaria transmission in tropical forests. In respect of a hypothesis suggesting that non-human hosts may be reservoirs of malaria-parasites [15], the present model can be extended by means of new compartments along with theirs parameters representing the role of susceptible and infective primates. Moreover, the present model assumes that all host species have the same tolerance to mosquito bites. Considering that animals may have more tolerance to mosquito bites than humans, this assumption can be unlikely and thus dilution effect herein may predict a underestimated blocking-transmission impact because of (more) intolerant dead-end hosts. It is therefore important to evaluate how primates as *Plasmodium*-reservoirs and tolerance of warm-blooded animals to mosquito bites may affect, positive or negatively, dilution effect predictions in the dynamics of malaria transmission.

*Plasmodium*-infected *An. cruzii* were found within human domiciles during epidemics occurring in the municipalities of Blumenau, Brusque, Joinvile, and Florianópolis, all located within the Atlantic Forest region, in the 1940s and 1950s. One determinant of the malaria burden in those days was the rapid increase in the population of susceptible humans, which reached 800,000 in a short period of time [21]. Another determinant was that humans were immunologically naïve to *Plasmodium* species infection. Consequently, while clearing native forest for agriculture and cattle farming, they lived in the nearby jungle, which increased the contact between humans and infective mosquitoes. It is likely that more recent malaria epidemics in the Amazon Forest occurred because of ecological and social determinants similar to those present in the Machadinho settlement project in the state of Rondônia between 1984 and 1995. Castro et al. observed that the prevalence of malaria increased rapidly in the early stages of settlement and subsequently decayed, reaching a low level 11 years later, which represents the general pattern of frontier malaria in the Amazon [37]. One way of avoiding malaria epidemics in tropical regions (mainly in the Amazon) is clearing large areas of forest and rapidly establishing agriculture or farming in order to limit the exposure of new settlers to infective mosquito bites [37]. This is in consonance with the traditional approach of forest clearing used in the Atlantic Forest in the 1950s [21]. In contrast, the results of the approach taken in the present study suggest that biodiversity contributes to disease control and thus ecosystems in tropical forests can be managed to sustain an equilibrium between high levels of biodiversity and the over-encroachment of human populations. Furthermore, diffuse mosquito vector competition can be considered a novel measure of vector control, especially because some *Anopheles* vector species seem not to be susceptible to indoor residual insecticide spraying and treated bed nets, which are currently the most successful strategies in Africa [8].

Contrary to what has long been believed, forest conservation and malaria control are not incompatible, and biodiversity issues should be included in the World Health Organization Malaria Eradication Research Agenda in order to achieve the desirable goals of biological conservation and maintenance of low malaria endemicity. Although releasing non-vector mosquitoes is not a practical alternative as vector control, conservation of the natural ecosystems may hinder transmission of malaria-parasites. The main application of the present model is to provide a formal framework in which biodiversity conservation and control of the human population size in protected areas are measures that can be taken to control transmission in any malarial endemic settings. The effect of mosquito vector diffuse competition means that policies of removal of native vegetation to eliminate malarial vectors, which were practiced in the past [21], have their shortcomings because they may also decrease non-vector community that buffers malarial transmission. For rural malaria, which includes *Anopheles gambiae* malarial dynamics in Africa, the mosquito vector diffuse competition is also a plausible underlying mechanism because it supports high transmission rates when native fauna is locally depleted by forest removal. Dead-end parasite transmission (dilution effect), by the framework herein proposed, was shown to be highly dependent on host tolerance. Consequently, there are two general predicted scenarios, i.e., 1) this mechanism may favour parasite decrease if the most tolerant host is a dead-end and 2) it may increase the vector population if tolerant hosts are present. It is noteworthy that these scenarios are not mutually exclusive. According to the subliminal message in Smith and colleagues' work [63], scientists of the present century should go beyond the Ross-Macdonald's Theory in order to have better insights on the ways that make possible the control of malarial transmission. In addition, the present model also makes qualitative predictions, and not just a correction in the value of 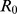, that are very distinct from the Ross-Macdonald (R-M) model, e.g., the behavior of 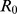 when 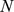 (i.e., human population) increases: it decreases in the R-M model, but it increases in the dynamics of the present model because greater 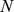 implies higher vector-host contacts, leading to increase of parasite dissemination. The present model constitutes an essential step for understanding the dynamics of malaria transmission in tropical forest ecosystems that can provide the service of hindering malaria epidemics, allowing to reconcile malaria control with conservation of biodiversity.

## Supporting Information

Text S1 **Collected data and data from the literature regarding estimates of input parameters utilized in the mathematical model of malaria transmission**.
(PDF)

Text S2
**Explicit derivation of the basic reproduction number** 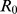 **.**
(PDF)

Text S3 **Analysis of an alternative model.**
(PDF)

Figure S1
***Plasmodium vivax*** **'s presence in the immediate surrounding region of Parque Estadual da Ilha do Cardoso.** Curado and others [13] has found positivity of IgG antibodies against *P. vivax* in human samples from Iporanga municipality (prevalence 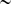
50%). Castro Duarte and others [15] detected *Plasmodium vivax* infections in howler-monkeys (i.e., *Alouatta guariba clamitans*) from the Atlantic Forest (possibly Juquitiba municipality) (prevalence 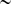
6%). Finally, D'Avila Couto and others [17] estimated that near 400 cases of malaria (being 97.2% attributable to *P. vivax*) were confirmed between 1980 and 2007 by official agencies of epidemiological surveillance (e.g., Superintendência de Controle de Endemias da Secretaria de Estado da Saúde de São Paulo and Sistema de Informação de Agravos de Notificação).(PDF)

Figure S2
**Relationships between successes and attempts in mosquito biting events in a given day.** The X axis is the total number of mosquitoes (*An. cruzii*) (M) and non-vectors species (C). The Y axis is the total number of biting successes per day (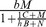). Guarani, The Guarani Mbya village; and Marujá, Marujá.(PDF)

Figure S3 **Human population and its geographical location.** Clear-cut areas in the northern part of The Guarani Mbya village (G) represent logged forest that are utilized to agriculture. In slopes of the southern part of The Guarani Mbya village (G) vertebrate animals can be hunted. Fishermen build houses for their families in Marujá (M) which are also utilized as hostels for ecotourists. Source: Instituto Florestal do Estado de São Paulo [64].
(PDF)

Figure S4 **Occurrence of mammals in the Parque Estadual da Ilha do Cardoso.** Mammal species were either seen or heard. Footprints were also utilized to indicate their presence. Legend: filled black circle, *Alouatta guariba* (howler monkey); hollow circle, *Mazama americana* (deer); hollow circle with vertical line, *Nasua nasua* (coati); filled black square, *Pecari tajacu* (collared peccari); hollow square, *Leopardus pardalis*, *L. wiedii* e *Herpailurus yaguarondi* (small spotted cats); hollow square with vertical line, *Sciurus ingrami* (squirrel); filled black triangle, *Cerdocyon thous* (fox); hollow triangle, *Eira barbara* (tayra); hollow triangle with vertical line, *Tayassu pecari* (white-lipped pecary); cross, *Dasyprocta leporina* (agouti). Source: Bernardo [45].
(PDF)

Figure S5 **Occurrence of birds in the Parque Estadual da Ilha do Cardoso.** Bird species were either seen or heard. Legend: filled black circle, *Ramphastos dicolorus* and *R. vitellinus* (toucans); hollow circle, *Penelope obscura* and *P. superciliaris* (guans); filled black square, *Pipile jacutinga* (guan); hollow square, *Crypturellus obsoletus* (tinamou); filled black triangle, *Odontophorus capueira* (spot-winged wood quail); hollow triangle, *Tinamus solitarius* (tinamou). Source: Bernardo [45].
(PDF)

Figure S6
**Vegetation and altitude at sampling sites of non-vector mosquito species (** 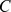 **) and** ***Anopheles cruzii*** **(** 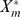 **): interpolations of ecologic niche axes.** A: Vegetation biomass (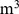 of wood per 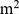); B: Altitude (meters above the sea). Points represent field sampling locations that were utilized for performing interpolations (grid of 200 m-spatial resolution). Source: Bernardi et al. [46].(PDF)

Figure S7
**Abundance of non-vector mosquito species (** 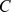 **) and** ***Anopheles cruzii*** **(** 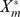 **): spatial abundance distribution modelling.** A: Abundance of *An. cruzii* (altitude 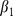 of 6.65 and vegetation biomass 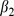 of 2.13; 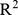-adjusted = 0.91); B: Abundance of *Ae. serratus* (vegetation biomass 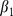 of 2.13; 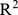-adjusted = 0.16); C: Abundance of *Li. durhami* (altitude 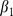 of 2.88 and vegetation biomass 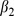 of 1.00; 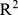-adjusted = 0.93); D: Abundance of *Ru. reversa* (altitude 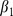 of 6.4; 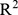-adjusted = 0.25); and E: Abundance of *Wy. quasilongirostris* (altitude 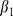 of 5.5; 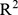-adjusted = 0.34; grid of 200 m-spatial resolution). G, The Guarani Mbya village; and M, Marujá.(PDF)

Figure S8
**Sensitivity analysis: if** 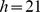 **then** 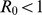 **.** A, B: Decrease in abundance of non-vector mosquito species can increase risk of malaria transmission (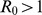) in The Guarani Mbya village and Marujá, respectively; C, D: Decrease in abundance of non-host vertebrate species does not increase risk of malaria transmission (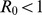) in The Guarani Mbya village and Marujá, respectively. The parameter 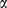 is 5.3 in The Guarani Mbya village and 3 in Marujá.(PDF)

Figure S9
**Sensitivity analysis: if** 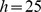 **then** 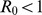 **.** A, B: Decrease in abundance of non-vector mosquito species can increase risk of malaria transmission (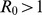) in The Guarani Mbya village and Marujá, respectively; C, D: Decrease in abundance of non-host vertebrate species does not increase risk of malaria transmission (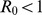) in The Guarani Mbya village and Marujá, respectively. The parameter 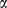 is 4.7 in The Guarani Mbya village and 2.8 in Marujá.(PDF)

Figure S10
**Sensitivity analysis: if** 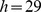 **then** 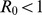 **.** A, B: Decrease in abundance of non-vector mosquito species can increase risk of malaria transmission (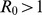) in The Guarani Mbya village and Marujá, respectively; C, D: Decrease in abundance of non-host vertebrate species does not increase risk of malaria transmission (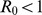) in The Guarani Mbya village and Marujá, respectively. D: Increase in abundance of non-host vertebrate species can increase risk of malaria transmission (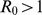) in Marujá, which is supported in the work by Saul [28]. The parameter 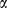 is 4.3 in The Guarani Mbya village and 2.6 in Marujá.(PDF)

Figure S11
**Basic reproduction number (** 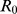 **) as a function of the human population size (** 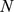 **), for the three models compared.** The other parameter models are the same from Table 1 (main text) for the Marujá.(PDF)

Table S1 **Animal and bird species, density and population size estimates in the Parque Estadual da Ilha do Cardoso.**
(PDF)

Table S2 **Mosquito species and vegetation types in the Parque Estadual da Ilha do Cardoso.**
(PDF)

Table S3 **Mosquito abundance regression models, independent variables and Akaike Information Criteria values.**
(PDF)
