# Supplementary material for: Biodiversity Can Help Prevent Malaria Outbreaks in Tropical Forests
Source: PLoS Negl Trop Dis. 2013 Mar 21;7(3):e2139. doi: 10.1371/journal.pntd.0002139 (PMC3605282; doi:10.1371/journal.pntd.0002139)
Supplement: Text S1 — Collected data and data from the literature regarding estimates of input parameters utilized in the mathematical model of malaria transmission. (PDF) [file pntd.0002139.s001.pdf]

1 Introduction

Here, we better describe Materials and Methods section regarding estimates of input parameters which were utilized in the mathematical model of malaria transmission.

1.1 Human population size (N)

The management plan of Parque Estadual da Ilha do Cardoso [1] was utilized to estimate population size in The Guarani Mbya village and Marujá. Using Aerophotogrametric images (Figure S3), it was possible to identify the geographical localities where inhabitants logged to build houses or to cultivate crops. The Guarani Mbya village is in the northwest, where approximately 150 people live today [2]. They can log natural vegetation on lowlands for small-scale agriculture and to hunt animals in slopes of the primary tropical forest (Figure S3). Marujá is located on the margins of the southern coastal plain (Figure S3), where 165 inhabitants subsist by fishing and exploring tourism [1].

1.2 Abundance of wild warm-blooded animals (B)

Christine São Bernardo and her advisor Dr. Galetti estimated the density of birds and mammals in Parque Estadual da Ilha do Cardoso (Table S1 [2]). Animal observations were performed in up to 273 km of transects in 13 trails throughout the region (Figure S4, Figure S5 [2]). Bird and mammal species density [2] was multiplied by the area of The Guarani Mbya village (2.8 km^2; Figure S3) and Marujá (0.8 km^2; Figure S3). As a result, estimates of abundance of bird and mammal species were 172 and 47, respectively (Table S1).

1.3 Abundance of non-vector mosquito species (C) and *Anopheles cruzii* (X_m^*)

Mosquito species that have overlapping period of biting activity were collected employing CDC-CO_2 traps [3] from 600 am to 1800 pm in altitudinal and vegetational succession gradients. Species identifications were based on the morphological identification keys [4–8]. As a result, a total of 3,299 mosquitoes belonging to 41 species were collected. *Plasmodium* vector *Anopheles cruzii* and non-vectors *Ae. serratus*, *Li. durhami*, *Ru. reversa*, and *Wy. quasilongirostris* were found in restinga and forest (Table S2), where females may compete for blood sources, such as agoutis in the former, and howler monkeys and squirrels in the latter. It was assumed that mosquito blood-feeding relied primarily on host availability [9].

Variations along the altitudinal and vegetational succession gradients were assumed to provide specific resting places after blood-feeding to the egg maturation, which could influence both *An. cruzii* and non-vector species abundances. Altitude and vegetation biomass data were obtained in locations thoroughly sampled [10] and then a generalized linear Gaussian geostatistical model with Bayesian inference was run to interpolate the altitude and vegetation biomass in the statistical environment R 2.13 with geoR package [11]. Results are shown in Figure S6A, in which light green represents scrubs on plains and hilltops, whereas dark green means tropical pluvial forest. In Figure S6B, lowlands were represented as light brown (<50 m) and hillsides as dark brown (from 50 to 350 m), whereas hilltops were underrepresented.

Abundance of *An. cruzii* and non-vectors species was, firstly, regressed against values of altitude and vegetation biomass interpolations and, secondly, extrapolated to Parque Estadual da Ilha do Cardoso (Figure S7A-E), based on the best fitted and parsimonious regression model [12, 13] (Table S3). Zonal statistic in the Spatial Analyst extension of ArcMap (www.esri.com) was utilized to estimate abundances of *An. cruzii* (X_m^*) as 300 and non-vectors (C) as 3,640 in Marujá and 1,514 (*An. cruzii*, X_m^*) and 14,101 (non-vectors, C) in The Guarani Mbya village.

1.4 *Anopheles cruzii* biting rate (b) and mortality rate (μ)

Roseli La Corte dos Santos and her advisor Dr. Forattini estimated the vectorial capacity of mosquitoes of subgenera *Kerteszia* of *Anopheles* in Atlantic Forest, southeastern Brazil [14].

Roseli considered the gonotrophic cycle estimate as approximately 4 days under laboratory conditions [14]. In this laboratory experiment *An. cruzii* female cohorts were accompanied from blood-feeding until oviposition giving an estimate of the length of a physiological gonotrophic cycle. Another couple of scientists also performed a similar experiment with *An. cruzii* female cohorts and estimated duration of gonotrophic cycle as 4.01 days [15], which means that the gonotrophic cycle estimate as 4 days is reasonable and also concensus in the literature. Following, some authors consider that *An. cruzii* has gonotrophic discordance [14, 16], and then it was assumed that females could bite two times in average per cycle duration (4 days), which thus led to an estimate of biting rate of 0.5 bites/day (i.e., $\frac{\text{number of bites per cycle}}{\text{gonotrophic cycle duration in days}} = \frac{2}{4} = 0.5$ bite/female/day).

Roseli considered the daily survival estimate as 0.45 employing mark-release-recapture experiments [14]. This value was obtained from a regression analysis (Milby and Reisen [17]) of marked-and-recaptured females in function of days after the day of release of females (y = -0.7958x + 5.3103; $R^2_{\text{multiple}} = 0.7612$; exp(-0.7958) = 0.4512201 = daily survival). It was considered that *An. cruzii* mortality rate was independent of density, what amounts to say that the mortality rate (μ) is related to the daily survival (s_{day}) by $\mu = -\log(s_{day})$, providing the value of $\mu = -\log(0.45) = 0.8$/day. Moreover, we calculated that, with a vector mortality rate as 0.8 per day, 0.17% of the female population will remain alive until the 8th day (when *Plasmodium vivax* extrinsic period is complete [18]), being 24 females in the Guarani Mbya tribe and 6 in Marujá. It is well-known that only few females of the population can survive long enough to become infective and thus our model estimate has connection with the real nature of this parameter.

1.5 *Anopheles cruzii* conversion rate (α)

Anopheles cruzii was present, so it must be true that emergence of *An. cruzii* adults in average surpasses its mortality rate (i.e., $\alpha b > \mu$), and at the same time abundance of non-vector mosquito species are not high enough to competitively exclude *An. cruzii* (i.e., $C < \left(\frac{\alpha b}{\mu} - 1\right) h(B+N)$). Equilibrium population between *An. cruzii* and non-vector mosquito species was derived from the model equations, and is given by:

$$X_m^* = \left(\frac{\alpha b}{\mu} - 1\right) h(B+N) - C\,. \tag{1}$$

Note that when $\frac{\alpha b}{\mu} \approx 1$ *An. cruzii* is not present because of the lack of environmental conditions that offer niche to the species. For example, dune pioneering vegetation ecosystem represents a situation in where *An. cruzii* niche requirement is absent because of the lack of bromeliads in where its larvae develop. In this situation, α is, probably, under 1 and $\frac{\alpha b}{\mu}$ may be < 1. However, in our study the Guarani Mbya tribe is in a forest and Marujá is in a "restinga" vegetation, in where *An. cruzii* is present (Table S2).

It was considered that the estimated populations of mosquitoes (see Section 1.3) are in fact equilibrium ones, and we used those values to find out the conversion rate α in the wild. Using input parameters discussed in the previous subsections in the formula above, we have:

$$1,514 = \left(\frac{\alpha\, 0.5}{0.8} - 1\right) 20(172 + 150) - 14,101 \Rightarrow \alpha = 5.5$$

for The Guarani Mbya village and

$$300 = \left(\frac{\alpha\, 0.5}{0.8} - 1\right) 20(47 + 165) - 3,640 \Rightarrow \alpha = 3.1$$

in Marujá.

The parameter α was higher (5.5) in The Guarani Mbya village than in Marujá (3.1) because the former place is in a forest and the latter is in a "restinga" vegetation. Veloso and others [19] performed an intensive ecological study of larval habitats of *An. cruzii* in Atlantic Forest and observed that this species is more associated with forest environment than with "restinga" vegetation. The α parameter estimate represents environmental conditions (mainly associated with larval habitats), and not simply a physiological characteristic.

1.6 Host tolerance (h)

Success to feed upon a host was assumed to be determined by the lack of host defensive responses to biting mosquitoes, which is supported by the works of Kelly [20] and Edman and others [21]. In the present work, host tolerance (h) is a phenomenological parameter, providing a functional response of hosts to mosquito density. This functional response represents here the simplest model for a phenomenon that increases linearly when mosquito density is low but reaches a saturation point when mosquito density is high (Figure S2). In addition, h is an adimensional parameter representing simply the number of bites. To estimate host tolerance, however, we need the number of bites received before starting a defensive behavior. Tolerance (i.e., number of bites per day without a host defensive behavior) is herein named as θ which equals 10 (bites / day), a common sense number representing how many times a given host (e.g., humans in a forest) is not bothered about being bitten during mosquito haematophagic activity:

$$Y_h^{BITES} = \frac{BITES * Y_h}{(B+N)} \tag{2}$$

where Y_h^{BITES} is the number of bites upon infected humans, $B+N$ is the total number of hosts, and $BITES$ is the total number of bites which is in function of

$$BITES = bX_m SUCCESS \tag{3}$$

where b is the biting rate, X_m is the number of *Anopheles cruzii* and $SUCCESS$ is the success factor of mosquito versus host, i.e., being mathematically

$$SUCCESS = \left[1 + \frac{bC + bM}{\theta B + \theta N}\right]^{-1} = \left[1 + \frac{1}{h}\frac{C+M}{B+N}\right]^{-1} \tag{4}$$

where $h = \frac{\theta}{b}$, $(bC + bM)$ is the total number of attempts of biting mosquitoes upon hosts per a given time, and $(\theta B + \theta N)$ is the total number of bites in which hosts can repel 50% of attempts of biting mosquitoes per a given time. It is important to note that the success factor (i.e., SUCCESS) must have the following properties:

- goes to 1 when $M = C = 0$ or when $N + B \to +\infty$
- strictly, decreases with M and C and increases with N and B
- goes to 0 when $M \to +\infty$ or $N, B \to 0$ (because nobody can take infinite bites)

Host tolerance (h) may be interpreted as an order-of-magnitude estimate, being related to *real* parameters (i.e., possible to be estimated in laboratory or field experiments) such as tolerance (θ) and biting rate (b). Moreover, it is clear that we made two simplifications: 1) tolerances were assumed to be equal for both hosts, i.e., B and N, and 2) biting rates rates were assumed to be equal for all mosquitoes, i.e., C and M. Although these assumptions may be strong, they permitted us to consider *An. cruzii*'s b and humans' θ estimates as proxies for other species.

As the number of bites in a day without a host defensive behavior was estimated as 10 and *An cruzii* biting rate (0.5), then h parameter equals 20 (i.e., $h = \frac{10}{0.5} = 20$). Total biting success per day is a Power-based function and, therefore, it decays up to its asymptote (Figure S2).

Since this value ($h = 20$) is harder to establish with precision, we performed a sensitivity analysis of malaria model to host tolerance (h), in which h was varied keeping $M = X_m^*$ constant and thus α was adjusted, being possible to estimate a new R_0 value. For values of h smaller than 18, the R_0 was < 0 in both human settlements, so this is the minimum of the considered interval. The maximum value was chosen to be 30, which corresponds to a human tolerance far greater than observed. Thus mosquito vector diffuse competition and dead-end parasite transmission patterns were assessed for h values within 20 and 30 (e.g., 21, 25, and 29). As a result, no qualitative changes in the first interpretations (see the main text) could be made (Figure S8A-D, Figure S9A-D, and Figure S10A-D).

References

1. Instituto Florestal do Estado de São Paulo (1998) Plano de gestão ambiental do Parque Estadual da Ilha do Cardoso. São Paulo: Secretaria do Meio Ambiente. 47 p.

2. Bernardo CSS (2004) Abundância, densidade e tamanho populacional de aves e mamíferos cinegéticos no Parque Estadual Ilha do Cardoso, SP, Brasil. Piracicaba: Universidade de São Paulo [Master's thesis]. 156 p.

3. Laporta GZ, Sallum MAM (2011) Effect of CO_2 and 1-octen-3-ol attractants for estimating species richness and the abundance of diurnal mosquitoes in the southeastern Atlantic forest, Brazil. Mem Inst Oswaldo Cruz 106: 279-284.

4. Lane J (1953) Neotropical Culicidae. São Paulo: EDUSP. 1112 p.

5. Correa RR, Ramalho GR (1956) Revisão de *Phoniomyia* Theobald, 1903 (Diptera, Culicidae, Sabethini). Folia Clin Biol 25: 1-176.

6. Harbach RE (1994) The subgenus *Sabethinus* of *Sabethes* (Diptera: Culicidae). Syst Entomol 19: 207-234.

7. Forattini OP (2002) Culicidologia médica. São Paulo: EDUSP. 860 p.

8. Sallum MAM, Santos CLS, Wilkerson RC (2009) Studies on *Anopheles* (*Kerteszia*) *homunculus* Komp (Diptera: Culicidae). Zootaxa 2299: 1-18.

9. Chaves LF, Harrington LC, Keogh CL, Nguyen AM, Kitron UD (2010) Blood feeding patterns of mosquitoes: random or structured? Front Zool 7.

10. Bernardi JVE, Landim PMB, Barreto CL, Monteiro RC (2005) Spatial study of the vegetation gradient from Cardoso Island State Park, SP, Brazil. Holos Environ 5: 1-21.

11. Diggle PJ, Ribeiro Jr PJ (2007) Model-based geostatistics. New York: Springer. 228 p.

12. Pinheiro J, Bates D, DebRoy S, Sarkar D (2011) nlme: Linear and Nonlinear Mixed Effects Models. Vienna: R Development Core Team, R package version 3.1-101.

13. Wood SN (2011) Fast stable restricted maximum likelihood and marginal likelihood estimation of semiparametric generalized linear models. J R Stat Soc Series B Stat Methodol 73: 3-36.

14. Santos RLC (2001) Medida da capacidade vetorial de *Anopheles albitarsis* e de *Anopheles* (*Kerteszia*) no Vale do Ribeira, São Paulo. São Paulo: Universidade de São Paulo [Ph.D. thesis]. 81 p.

15. Chahad-Ehlers S, Lozovei AL, Marques MD (2007) Reproductive and post-embryonic daily rhythm patterns of the malaria vector *Anopheles* (*Kerteszia*) *cruzii*: aspects of the life cycle. Chronobiol Int 24: 289–304.

16. Forattini OP, Kakitani I, Massad E, Marucci D (1996) Studies on mosquitoes (Diptera: Culicidae) and anthropic environment: 11-Biting activity and blood-seeking parity of *Anopheles* (*Kerteszia*) in South-Eastern Brazil. Rev Saúde Pública 30: 107–114.

17. Milby MM, Reisen WK (1989) Estimation of vectorial capacity: vector survivorship. Bull Soc Vector Ecol 14: 47–54.

18. Russell RC (1987) Seasonal abundance, longevity and population age composition of potential malaria vectors in northern and southern australia. Aust J Zool 35: 289–306.

19. Veloso HP, Moura JV, Klein RM (1956) Delimitação ecológica dos anofelíneos do subgênero *Kerteszia* na região costeira do sul do Brasil. Mem Inst Oswaldo Cruz 54: 517–534.

20. Kelly DW (2001) Why are some people bitten more than others? Trends Parasitol 17: 578–581.

21. Edman JD, Webber LA, Schmid AA (1974) Effect of host defenses on the feeding pattern of *Culex nigripalpus* when offered a choice of blood sources. J Parasitol 60: 874–883.
