# Supplementary material for: Biodiversity Can Help Prevent Malaria Outbreaks in Tropical Forests
Source: PLoS Negl Trop Dis. 2013 Mar 21;7(3):e2139. doi: 10.1371/journal.pntd.0002139 (PMC3605282; doi:10.1371/journal.pntd.0002139)
Supplement: Text S2 — Explicit derivation of the basic reproduction number . (PDF) [file pntd.0002139.s002.pdf]

1 R_0 calculation

Below, we present a derivation of the expression for R_0 of the main model, following the method presented in [1]. The procedure for the Ross–MacDonald model is completely analogous.

We denote by X_m^* the population of vector mosquitoes in the absence of infection, as in the main text, and write down the matrices F and V, used to build the *next generation matrix* K:

$$F = \begin{pmatrix} 0 & \frac{bT_{hM}N}{(B+N)\left(1+\frac{1}{h}\frac{C+X_m^*}{B+N}\right)} \\ \frac{bT_{Mh}X_m^*}{(B+N)\left(1+\frac{1}{h}\frac{C+X_m^*}{B+N}\right)} & 0 \end{pmatrix}$$

$$V = \begin{pmatrix} \gamma & 0 \\ 0 & \mu \end{pmatrix}$$

$$K = FV^{-1} = \begin{pmatrix} 0 & \frac{1}{\mu}\frac{bT_{hM}N}{(B+N)\left(1+\frac{1}{h}\frac{C+X_m^*}{B+N}\right)} \\ \frac{1}{\gamma}\frac{bT_{Mh}X_m^*}{(B+N)\left(1+\frac{1}{h}\frac{C+X_m^*}{B+N}\right)} & 0 \end{pmatrix}$$

The value of R_0 will be given by the largest non-negative eigenvalue of the next generation matrix, which in this case is the same as the *spectral radius* ρ of the matrix K, as follow:

$$\begin{aligned} R_0 = \rho(K) &= \frac{b}{(B+N)\left(1+\frac{1}{h}\frac{C+X_m^*}{B+N}\right)}\sqrt{\frac{T_{hM}T_{Mh}NX_m^*}{\gamma\mu}} \\ &= \frac{\mu}{\alpha(B+N)}\sqrt{\frac{T_{hM}T_{Mh}NX_m^*}{\gamma\mu}} \\ &= \frac{\mu}{\alpha(B+N)}\sqrt{\frac{T_{hM}T_{Mh}N\left[\left(\frac{\alpha b}{\mu}-1\right)h(B+N)-C\right]}{\gamma\mu}} \end{aligned}$$

References

1. van den Driessche P, Watmough J (2008) Further Notes on the Basic Reproduction Number. In: Brauer F, van den Driessche P, Wu J, Allen LJS, editors. Mathematical epidemiology. Heidelberg: Springer-Verlag. pp. 159–178.
