# Supplementary material for: Biodiversity Can Help Prevent Malaria Outbreaks in Tropical Forests
Source: PLoS Negl Trop Dis. 2013 Mar 21;7(3):e2139. doi: 10.1371/journal.pntd.0002139 (PMC3605282; doi:10.1371/journal.pntd.0002139)
Supplement: Text S3 — Analysis of an alternative model. (PDF) [file pntd.0002139.s003.pdf]

1 Alternative model

In this section we briefly analyze a variant model from that of the main text, to make clearer what are the consequences of specific ecological hypotheses assumed.

The model described by equations (8-11, main text) differs from the Ross–MacDonald one by introducing two effects: a saturating biting rate, caused by the diffuse competition among mosquitoes for feeding bites; and a limitation of the vector mosquito population by number of bites, while other competing mosquito species are regulated by factors not included in the model. The diffuse competition effect is essential to our model, as it is thought to cause decrease in number of bites and thus malaria risk.

In order to separate these effects, we are going to relax the second one and assume instead a constant vector mosquito population. This variant model is given by the system of equations:

$$\frac{dX_h}{dt} = -\frac{bT_{hm}X_hY_m}{(B+N)\left(1+\frac{1}{h}\frac{C+M}{B+N}\right)} + \gamma Y_h \tag{1}$$

$$\frac{dY_h}{dt} = \frac{bT_{hm}X_hY_m}{(B+N)\left(1+\frac{1}{h}\frac{C+M}{B+N}\right)} - \gamma Y_h \tag{2}$$

$$\frac{dX_m}{dt} = \mu Y_m - \frac{bT_{mh}X_mY_h}{(B+N)\left(1+\frac{1}{h}\frac{C+M}{B+N}\right)} \tag{3}$$

$$\frac{dY_m}{dt} = \frac{bT_{mh}X_mY_h}{(B+N)\left(1+\frac{1}{h}\frac{C+M}{B+N}\right)} - \mu Y_m \ , \tag{4}$$

where $M = X_m + Y_m$.

Applying the same method from R_0 calculation (Text S2) one can calculate the basic reproduction number R_0 of this model, obtaining:

$$R_0 = \frac{b}{(B+N)\left(1+\frac{1}{h}\frac{C+X_m^*}{B+N}\right)}\sqrt{\frac{T_{hM}T_{Mh}NX_m^*}{\gamma\mu}} \ , \tag{5}$$

which is the same expression as before, but with the crucial difference that now X_m^* is given by the initial vector mosquito abundance, and does not vary with other model parameters or human abundance.

The main difference between this model and the main one is how the human population (N) influences the transmission dynamics. This can be seen in Figure S11 which shows that at low human population sizes R_0 increases with N for both main and alternative models (but not for Ross–MacDonald); and for larger populations alternative model gets close to Ross–MacDonald predictions, while main model increases and saturates at a higher value.

The inclusion of diffuse competition between mosquitoes decreases the risk of an outbreak of malaria at very low population sizes, which is intuitive and reasonable. On the contrary, Ross–MacDonald model predicts the opposite – which is to be expected since it allows each human to take several hundreds of bites per day in that situation.

At larger population sizes, the relevant assumption is that vector population size is regulated by diffuse competition, being dependent on the human population size. In the absence of this regulation, alternative model approaches the Ross–MacDonald predictions for large N, while main model (equations 8-11, main text) predicts a higher risk of outbreak of malaria as human population increases. Therefore, we chose the most conservative model (i.e., main model), which gives a pessimistic scenario for population sizes concerned herein.
