# Supplementary material for: Biodiversity Can Help Prevent Malaria Outbreaks in Tropical Forests
Source: PLoS Negl Trop Dis. 2013 Mar 21;7(3):e2139. doi: 10.1371/journal.pntd.0002139 (PMC3605282; doi:10.1371/journal.pntd.0002139)
Supplement: Figure S1 — Plasmodium vivax 's presence in the immediate surrounding region of Parque Estadual da Ilha do Cardoso. Curado and others [13] has found positivity of IgG antibodies against P. vivax in human samples from Iporanga municipality (prevalence 50%). Castro Duarte and others [15] detected Plasmodium vivax infections in howler-monkeys (i.e., Alouatta guariba clamitans) from the Atlantic Forest (possibly Juquitiba municipality) (prevalence 6%). Finally, D'Avila Couto and others [17] estimated that near 400 cases of malaria (being 97.2% attributable to P. vivax) were confirmed between 1980 and 2007 by official agencies of epidemiological surveillance (e.g., Superintendência de Controle de Endemias da Secretaria de Estado da Saúde de São Paulo and Sistema de Informação de Agravos de Notificação). (PDF) [file pntd.0002139.s004.pdf]

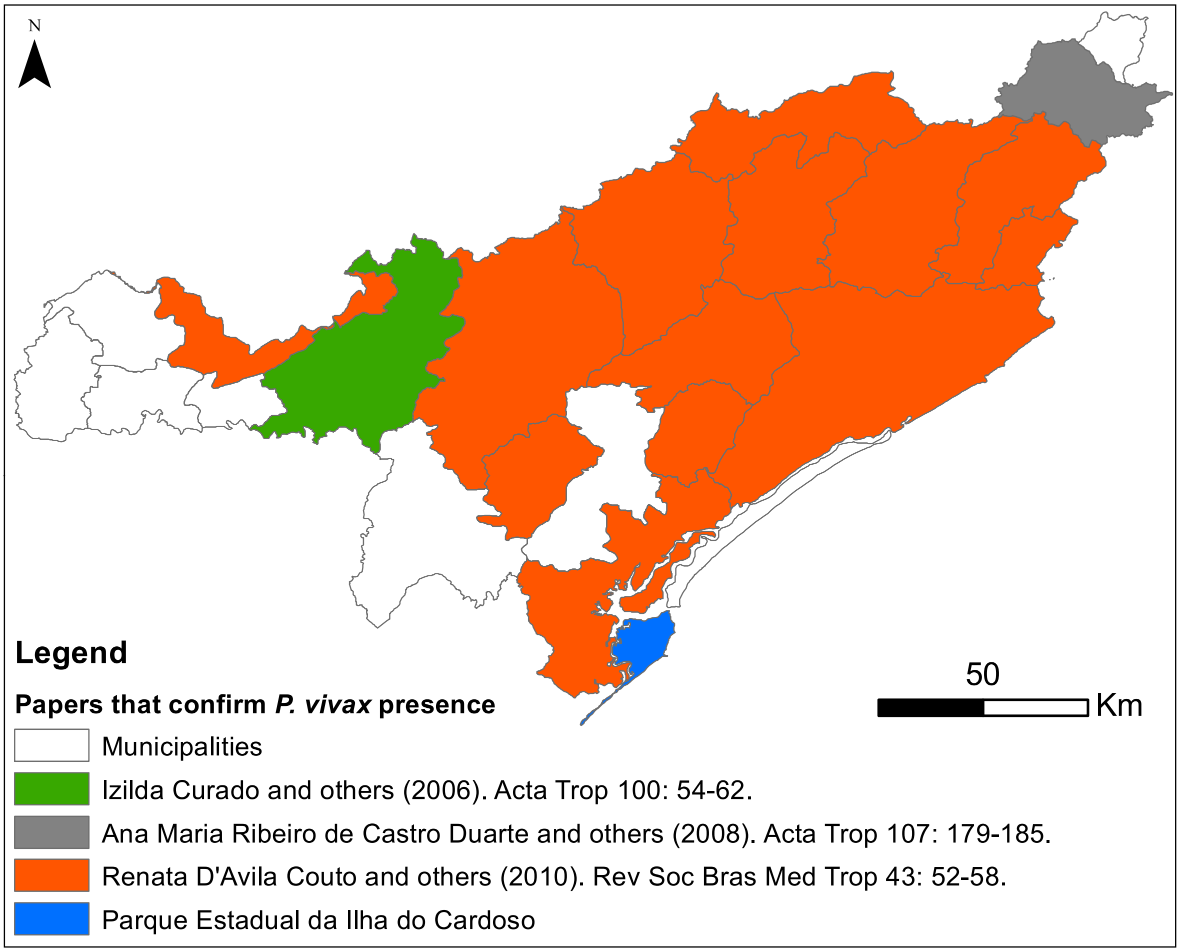

Figure S1. *Plasmodium vivax*'s presence in the immediate surrounding region of Parque Estadual da Ilha do Cardoso. Curado and others [1] has found positivity of IgG antibodies against *P. vivax* in human samples from Iporanga municipality (prevalence ∼ 50%). Castro Duarte and others [2] detected *Plasmodium vivax* infections in howler-monkeys (i.e., *Alouatta guariba clamitans*) from the Atlantic Forest (possibly Juquitiba municipality) (prevalence ∼ 6%). Finally, D'Avila Couto and others [3] estimated that near 400 cases of malaria (being 97.2 % attributable to *P. vivax*) were confirmed between 1980 and 2007 by official agencies of epidemiological surveillance (e.g., Superintendência de Controle de Endemias da Secretaria de Estado da Saúde de São Paulo and Sistema de Informação de Agravos de Notificação).

References

1. Curado I, Malafronte RS, Duarte AMRC, Kirchgatter K, Branquinho MS, et al. (2006) Malaria epidemiology in low-endemicity areas of the Atlantic Forest in the Vale do Ribeira, São Paulo, Brazil. Acta Trop 100: 54–62.

2. Duarte AMRC, Malafronte RS, Cerutti Jr C, Curado I, Paiva BR, et al. (2008) Natural *Plasmodium* infections in Brazilian wild monkeys: Reservoirs for human infections? Acta Trop 107: 179–185.

3. Couto RDA, Latorre MRD, Di Santi SM, Natal D (2010) Autochthonous malaria notified in the State of São Paulo: clinical and epidemiological characteristics from 1980 to 2007. Rev Soc Bras Med Trop 43: 52–58.
