# Supplementary material for: Biodiversity Can Help Prevent Malaria Outbreaks in Tropical Forests
Source: PLoS Negl Trop Dis. 2013 Mar 21;7(3):e2139. doi: 10.1371/journal.pntd.0002139 (PMC3605282; doi:10.1371/journal.pntd.0002139)
Supplement: Figure S2 — Relationships between successes and attempts in mosquito biting events in a given day. The X axis is the total number of mosquitoes (An. cruzii) (M) and non-vectors species (C). The Y axis is the total number of biting successes per day (). Guarani, The Guarani Mbya village; and Marujá, Marujá. (PDF) [file pntd.0002139.s005.pdf]

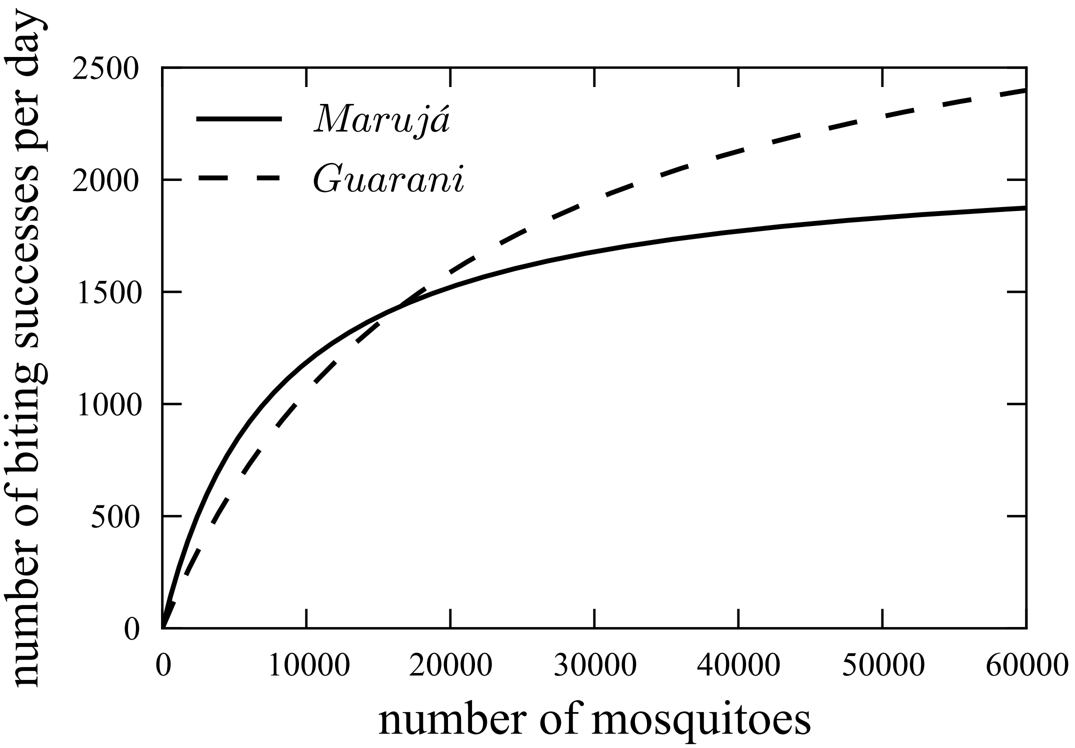

Figure S2. Relationships between successes and attempts in mosquito biting events in a given day. The X axis is the total number of mosquitoes (*An. cruzii*) (M) and non-vectors species (C). The Y axis is the total number of biting successes per day ($\frac{bM}{1+\frac{1}{h}\frac{C+M}{B+N}}$). Guarani, The Guarani Mbya village; and Marujá, Marujá.
