# Supplementary material for: Biodiversity Can Help Prevent Malaria Outbreaks in Tropical Forests
Source: PLoS Negl Trop Dis. 2013 Mar 21;7(3):e2139. doi: 10.1371/journal.pntd.0002139 (PMC3605282; doi:10.1371/journal.pntd.0002139)
Supplement: Figure S3 — Human population and its geographical location. Clear-cut areas in the northern part of The Guarani Mbya village (G) represent logged forest that are utilized to agriculture. In slopes of the southern part of The Guarani Mbya village (G) vertebrate animals can be hunted. Fishermen build houses for their families in Marujá (M) which are also utilized as hostels for ecotourists. Source: Instituto Florestal do Estado de São Paulo [64]. (PDF) [file pntd.0002139.s006.pdf]

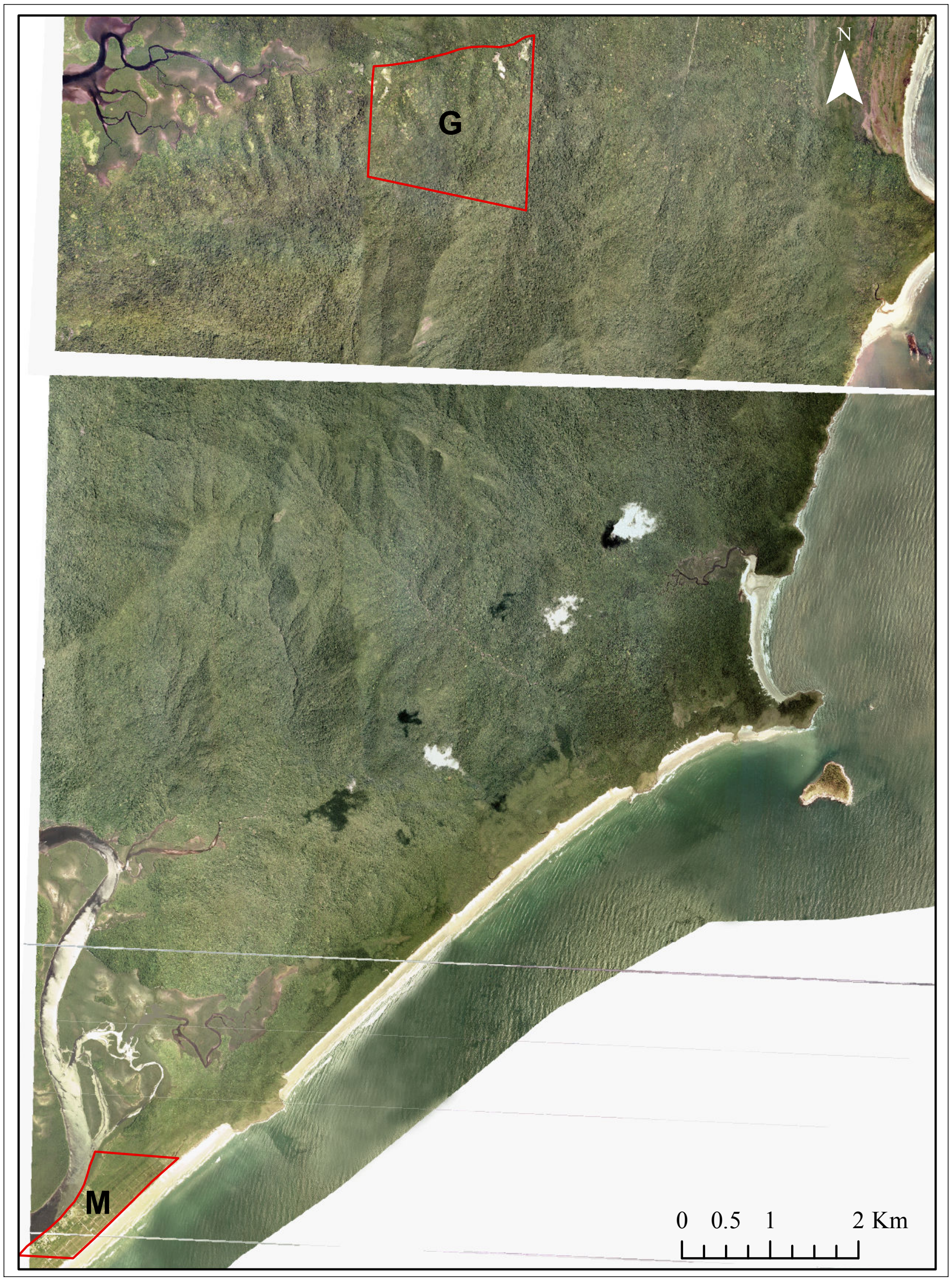

Figure S3. Human population and its geographical location. Clear-cut areas in the northern part of The Guarani Mbya village (G) represent logged forest that are utilized to agriculture. In slopes of the southern part of The Guarani Mbya village (G) vertebrate animals can be hunted. Fishermen build houses for their families in Marujá (M) which are also utilized as hostels for ecotourists. Source: Instituto Florestal do Estado de São Paulo [1].

References

1. Instituto Florestal do Estado de São Paulo (1998) Plano de gestão ambiental do Parque Estadual da Ilha do Cardoso. São Paulo: Secretaria do Meio Ambiente. 47 p.
