# Supplementary material for: Biodiversity Can Help Prevent Malaria Outbreaks in Tropical Forests
Source: PLoS Negl Trop Dis. 2013 Mar 21;7(3):e2139. doi: 10.1371/journal.pntd.0002139 (PMC3605282; doi:10.1371/journal.pntd.0002139)
Supplement: Figure S4 — Occurrence of mammals in the Parque Estadual da Ilha do Cardoso. Mammal species were either seen or heard. Footprints were also utilized to indicate their presence. Legend: filled black circle, Alouatta guariba (howler monkey); hollow circle, Mazama americana (deer); hollow circle with vertical line, Nasua nasua (coati); filled black square, Pecari tajacu (collared peccari); hollow square, Leopardus pardalis, L. wiedii e Herpailurus yaguarondi (small spotted cats); hollow square with vertical line, Sciurus ingrami (squirrel); filled black triangle, Cerdocyon thous (fox); hollow triangle, Eira barbara (tayra); hollow triangle with vertical line, Tayassu pecari (white-lipped pecary); cross, Dasyprocta leporina (agouti). Source: Bernardo [45]. (PDF) [file pntd.0002139.s007.pdf]

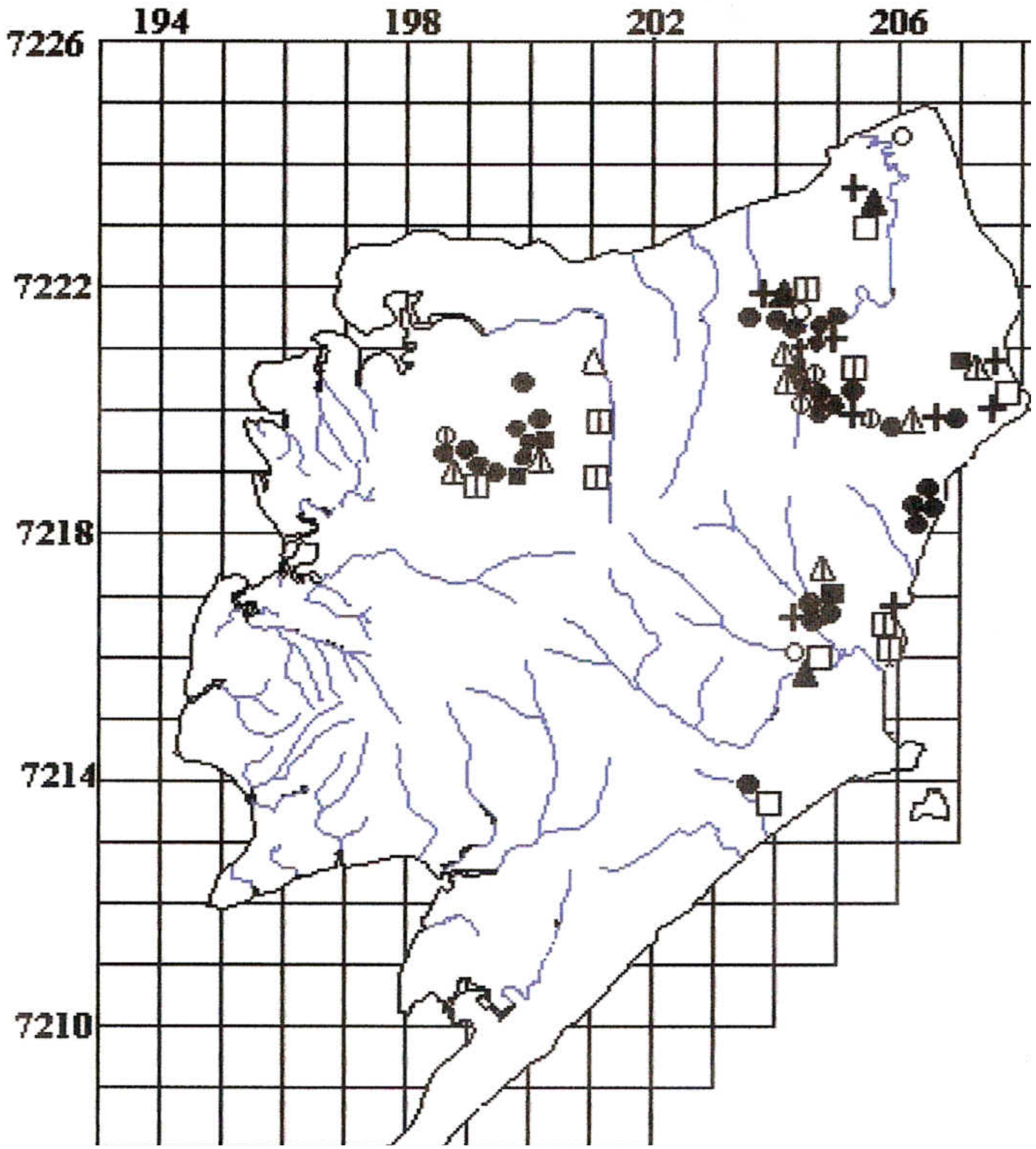

Figure S4. Occurrence of mammals in the Parque Estadual da Ilha do Cardoso. Mammal species were either seen or heard. Footprints were also utilized to indicate their presence. Legend: filled black circle, *Alouatta guariba* (howler monkey); hollow circle, *Mazama americana* (deer); hollow circle with vertical line, *Nasua nasua* (coati); filled black square, *Pecari tajacu* (collared peccari); hollow square, *Leopardus pardalis*, *L. wiedii* e *Herpailurus yaguarondi* (small spotted cats); hollow square with vertical line, *Sciurus ingrami* (squirrel); filled black triangle, *Cerdocyon thous* (fox); hollow triangle, *Eira barbara* (tayra); hollow triangle with vertical line, *Tayassu pecari* (white-lipped pecary); cross, *Dasyprocta leporina* (agouti). Source: Bernardo [1].

References

1. Bernardo CSS (2004) Abundância, densidade e tamanho populacional de aves e mamíferos cinegéticos no Parque Estadual Ilha do Cardoso, SP, Brasil. Piracicaba: Universidade de São Paulo [Master's thesis]. 156 p.
