# Supplementary material for: Biodiversity Can Help Prevent Malaria Outbreaks in Tropical Forests
Source: PLoS Negl Trop Dis. 2013 Mar 21;7(3):e2139. doi: 10.1371/journal.pntd.0002139 (PMC3605282; doi:10.1371/journal.pntd.0002139)
Supplement: Figure S5 — Occurrence of birds in the Parque Estadual da Ilha do Cardoso. Bird species were either seen or heard. Legend: filled black circle, Ramphastos dicolorus and R. vitellinus (toucans); hollow circle, Penelope obscura and P. superciliaris (guans); filled black square, Pipile jacutinga (guan); hollow square, Crypturellus obsoletus (tinamou); filled black triangle, Odontophorus capueira (spot-winged wood quail); hollow triangle, Tinamus solitarius (tinamou). Source: Bernardo [45]. (PDF) [file pntd.0002139.s008.pdf]

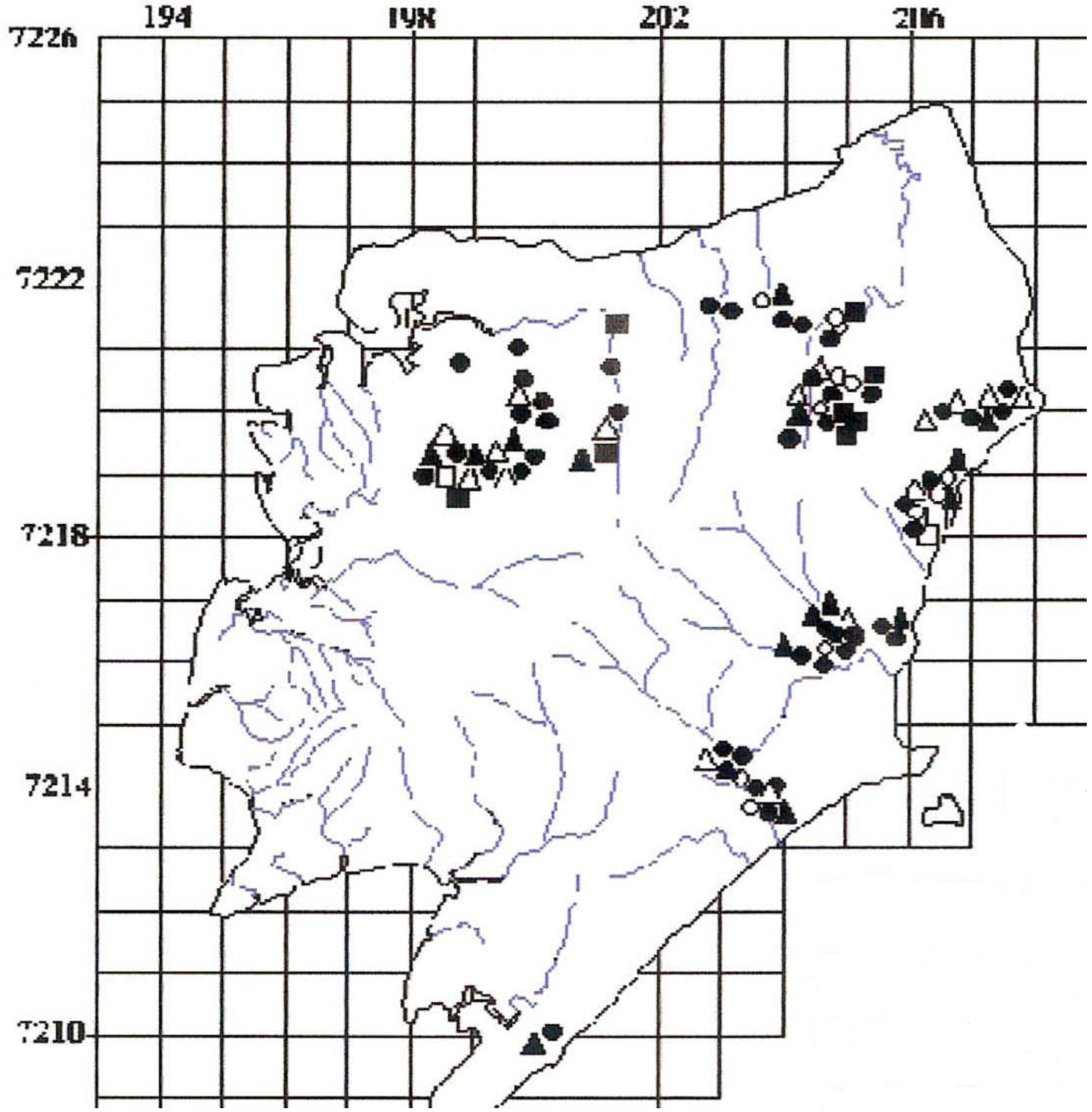

Figure S5. Occurrence of birds in the Parque Estadual da Ilha do Cardoso. Bird species were either seen or heard. Legend: filled black circle, *Ramphastos dicolorus* and *R. vitellinus* (toucans); hollow circle, *Penelope obscura* and *P. superciliaris* (guans); filled black square, *Pipile jacutinga* (guan); hollow square, *Crypturellus obsoletus* (tinamou); filled black triangle, *Odontophorus capueira* (spot-winged wood quail); hollow triangle, *Tinamus solitarius* (tinamou). Source: Bernardo [1].

References

1. Bernardo CSS (2004) Abundância, densidade e tamanho populacional de aves e mamíferos cinegéticos no Parque Estadual Ilha do Cardoso, SP, Brasil. Piracicaba: Universidade de São Paulo [Master's thesis]. 156 p.
