# Supplementary material for: Biodiversity Can Help Prevent Malaria Outbreaks in Tropical Forests
Source: PLoS Negl Trop Dis. 2013 Mar 21;7(3):e2139. doi: 10.1371/journal.pntd.0002139 (PMC3605282; doi:10.1371/journal.pntd.0002139)
Supplement: Figure S6 — Vegetation and altitude at sampling sites of non-vector mosquito species ( ) and Anopheles cruzii ( ): interpolations of ecologic niche axes. A: Vegetation biomass ( of wood per ); B: Altitude (meters above the sea). Points represent field sampling locations that were utilized for performing interpolations (grid of 200 m-spatial resolution). Source: Bernardi et al. [46]. (PDF) [file pntd.0002139.s009.pdf]

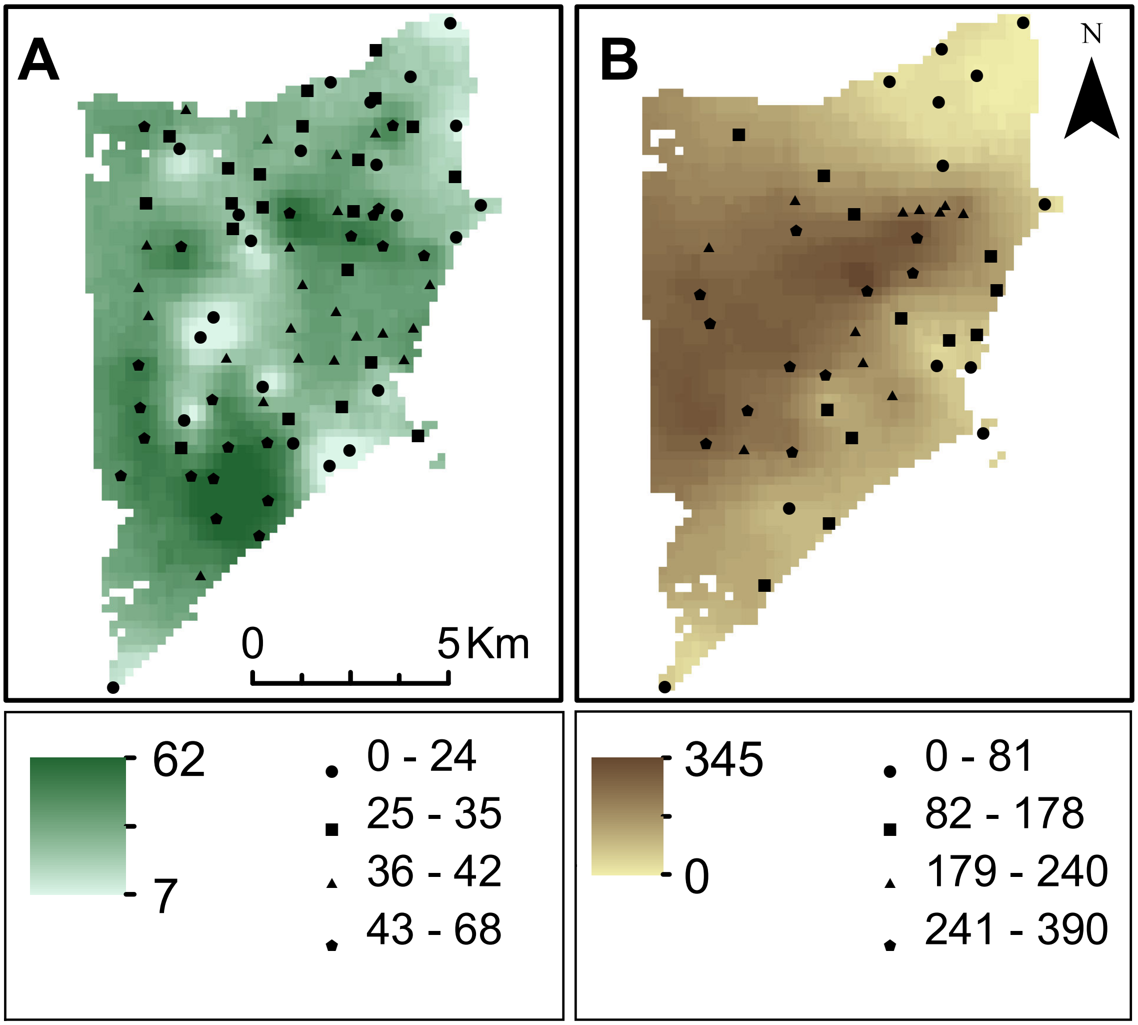

Figure S6. Vegetation and altitude at sampling sites of non-vector mosquito species (C) and *Anopheles cruzii* (X_m^*): interpolations of ecologic niche axes. A: Vegetation biomass (m^3 of wood per m^2); B: Altitude (meters above the sea). Points represent field sampling locations that were utilized for performing interpolations (grid of 200 m-spatial resolution). Source: Bernardi et al. [1].

References

1. Bernardi JVE, Landim PMB, Barreto CL, Monteiro RC (2005) Spatial study of the vegetation gradient from Cardoso Island State Park, SP, Brazil. Holos Environ 5: 1-21.
