# Supplementary material for: Biodiversity Can Help Prevent Malaria Outbreaks in Tropical Forests
Source: PLoS Negl Trop Dis. 2013 Mar 21;7(3):e2139. doi: 10.1371/journal.pntd.0002139 (PMC3605282; doi:10.1371/journal.pntd.0002139)
Supplement: Figure S7 — Abundance of non-vector mosquito species ( ) and Anopheles cruzii ( ): spatial abundance distribution modelling. A: Abundance of An. cruzii (altitude of 6.65 and vegetation biomass of 2.13; -adjusted = 0.91); B: Abundance of Ae. serratus (vegetation biomass of 2.13; -adjusted = 0.16); C: Abundance of Li. durhami (altitude of 2.88 and vegetation biomass of 1.00; -adjusted = 0.93); D: Abundance of Ru. reversa (altitude of 6.4; -adjusted = 0.25); and E: Abundance of Wy. quasilongirostris (altitude of 5.5; -adjusted = 0.34; grid of 200 m-spatial resolution). G, The Guarani Mbya village; and M, Marujá. (PDF) [file pntd.0002139.s010.pdf]

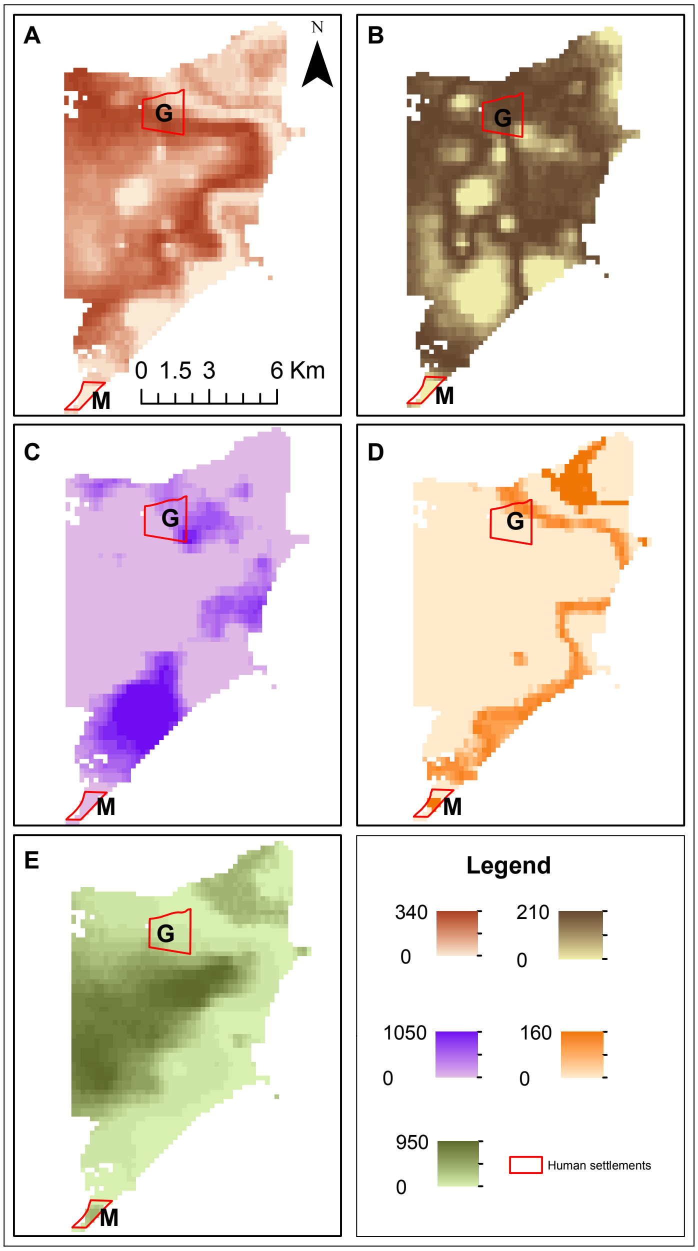

Figure S7. Abundance of non-vector mosquito species (C) and *Anopheles cruzii* (X_m^*): spatial abundance distribution modelling. A: Abundance of *An. cruzii* (altitude β_1 of 6.65 and vegetation biomass β_2 of 2.13; R^2-adjusted = 0.91); B: Abundance of *Ae. serratus* (vegetation biomass β_1 of 2.13; R^2-adjusted = 0.16); C: Abundance of *Li. durhami* (altitude β_1 of 2.88 and vegetation biomass β_2 of 1.00; R^2-adjusted = 0.93); D: Abundance of *Ru. reversa* (altitude β_1 of 6.4; R^2-adjusted = 0.25); and E: Abundance of *Wy. quasilongirostris* (altitude β_1 of 5.5; R^2-adjusted = 0.34; grid of 200 m-spatial resolution). G, The Guarani Mbya village; and M, Marujá.
