# Supplementary material for: Biodiversity Can Help Prevent Malaria Outbreaks in Tropical Forests
Source: PLoS Negl Trop Dis. 2013 Mar 21;7(3):e2139. doi: 10.1371/journal.pntd.0002139 (PMC3605282; doi:10.1371/journal.pntd.0002139)
Supplement: Figure S9 — Sensitivity analysis: if then . A, B: Decrease in abundance of non-vector mosquito species can increase risk of malaria transmission () in The Guarani Mbya village and Marujá, respectively; C, D: Decrease in abundance of non-host vertebrate species does not increase risk of malaria transmission () in The Guarani Mbya village and Marujá, respectively. The parameter is 4.7 in The Guarani Mbya village and 2.8 in Marujá. (PDF) [file pntd.0002139.s012.pdf]

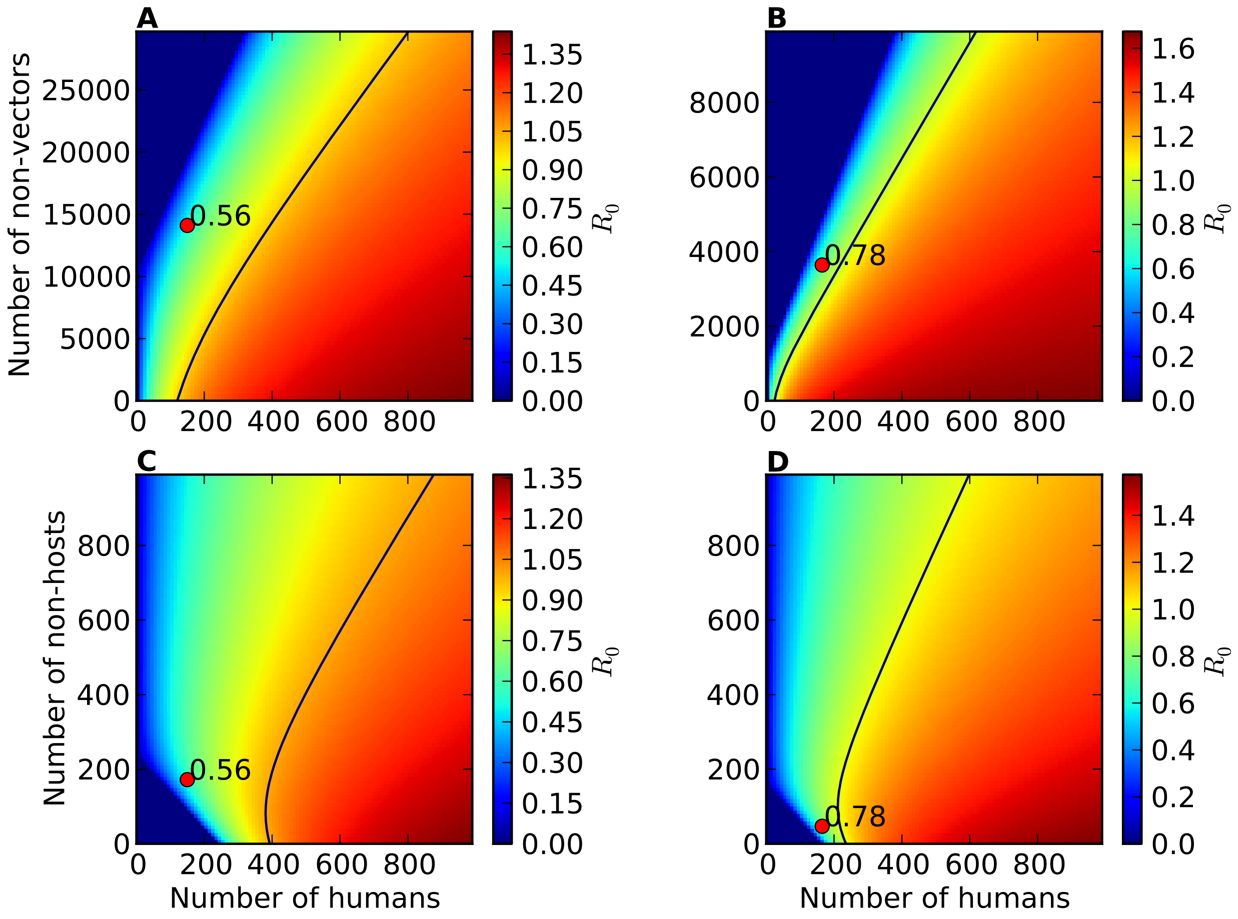


**Figure S9. Sensitivity analysis: if $h = 25$ then $R_0 < 1$.** A, B: Decrease in abundance of non-vector mosquito species can increase risk of malaria transmission ($R_0 > 1$) in The Guarani Mbya village and Marujá, respectively; C, D: Decrease in abundance of non-host vertebrate species does not increase risk of malaria transmission ($R_0 < 1$) in The Guarani Mbya village and Marujá, respectively. The parameter $\alpha$ is 4.7 in The Guarani Mbya village and 2.8 in Marujá.
