# Supplementary material for: Biodiversity Can Help Prevent Malaria Outbreaks in Tropical Forests
Source: PLoS Negl Trop Dis. 2013 Mar 21;7(3):e2139. doi: 10.1371/journal.pntd.0002139 (PMC3605282; doi:10.1371/journal.pntd.0002139)
Supplement: Figure S10 — Sensitivity analysis: if then . A, B: Decrease in abundance of non-vector mosquito species can increase risk of malaria transmission () in The Guarani Mbya village and Marujá, respectively; C, D: Decrease in abundance of non-host vertebrate species does not increase risk of malaria transmission () in The Guarani Mbya village and Marujá, respectively. D: Increase in abundance of non-host vertebrate species can increase risk of malaria transmission () in Marujá, which is supported in the work by Saul [28]. The parameter is 4.3 in The Guarani Mbya village and 2.6 in Marujá. (PDF) [file pntd.0002139.s013.pdf]

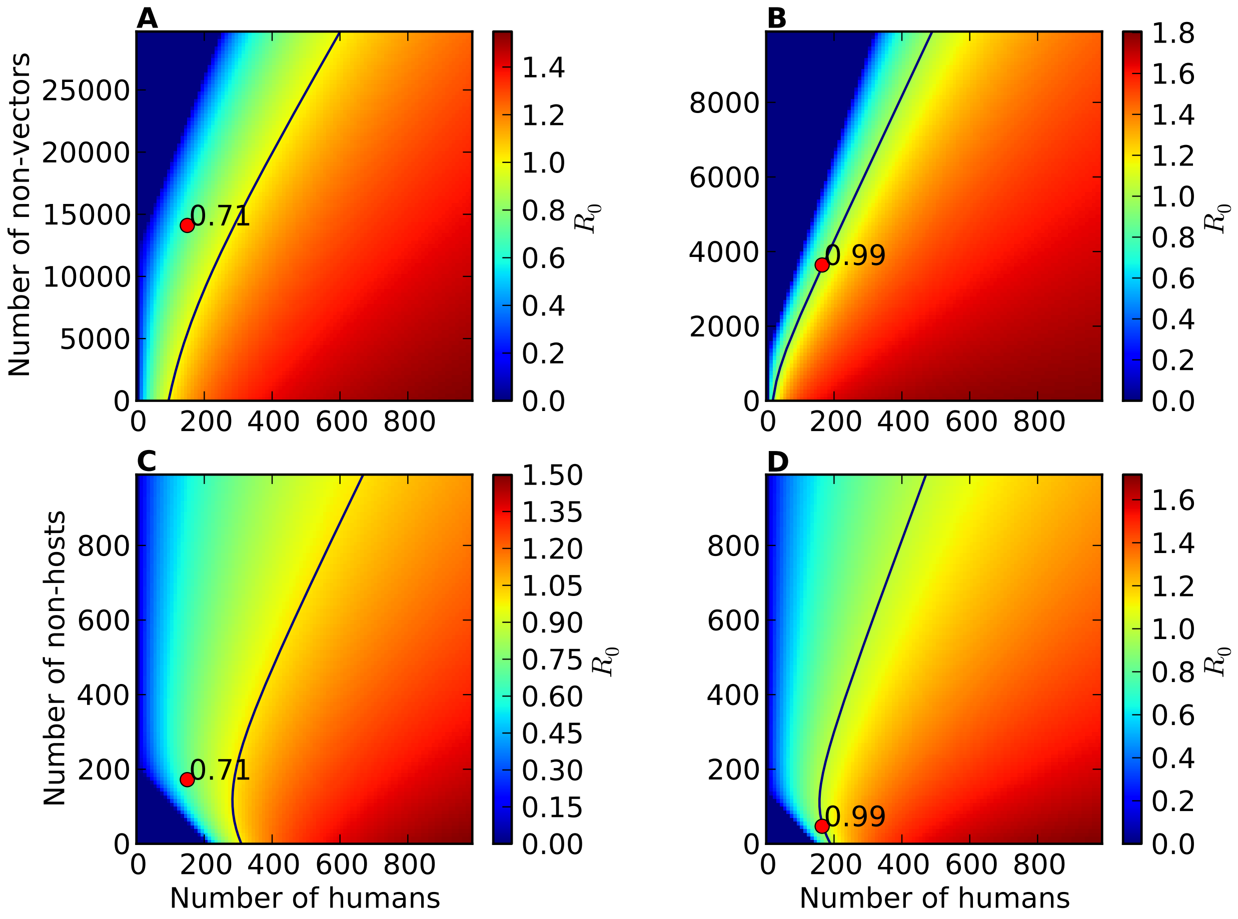

Figure S10. Sensitivity analysis: if $h = 29$ then $R_0 < 1$. A, B: Decrease in abundance of non-vector mosquito species can increase risk of malaria transmission ($R_0 > 1$) in The Guarani Mbya village and Marujá, respectively; C, D: Decrease in abundance of non-host vertebrate species does not increase risk of malaria transmission ($R_0 < 1$) in The Guarani Mbya village and Marujá, respectively. D: Increase in abundance of non-host vertebrate species can increase risk of malaria transmission ($R_0 > 1$) in Marujá, which is supported in the work by Saul [1]. The parameter α is 4.3 in The Guarani Mbya village and 2.6 in Marujá.

References

1. Saul A (2003) Zooprophylaxis or zoopotentiation: the outcome of introducing mortality while searching. Malaria J 2: 32.
