# Supplementary material for: Biodiversity Can Help Prevent Malaria Outbreaks in Tropical Forests
Source: PLoS Negl Trop Dis. 2013 Mar 21;7(3):e2139. doi: 10.1371/journal.pntd.0002139 (PMC3605282; doi:10.1371/journal.pntd.0002139)
Supplement: Figure S11 — Basic reproduction number ( ) as a function of the human population size ( ), for the three models compared. The other parameter models are the same from Table 1 (main text) for the Marujá. (PDF) [file pntd.0002139.s014.pdf]

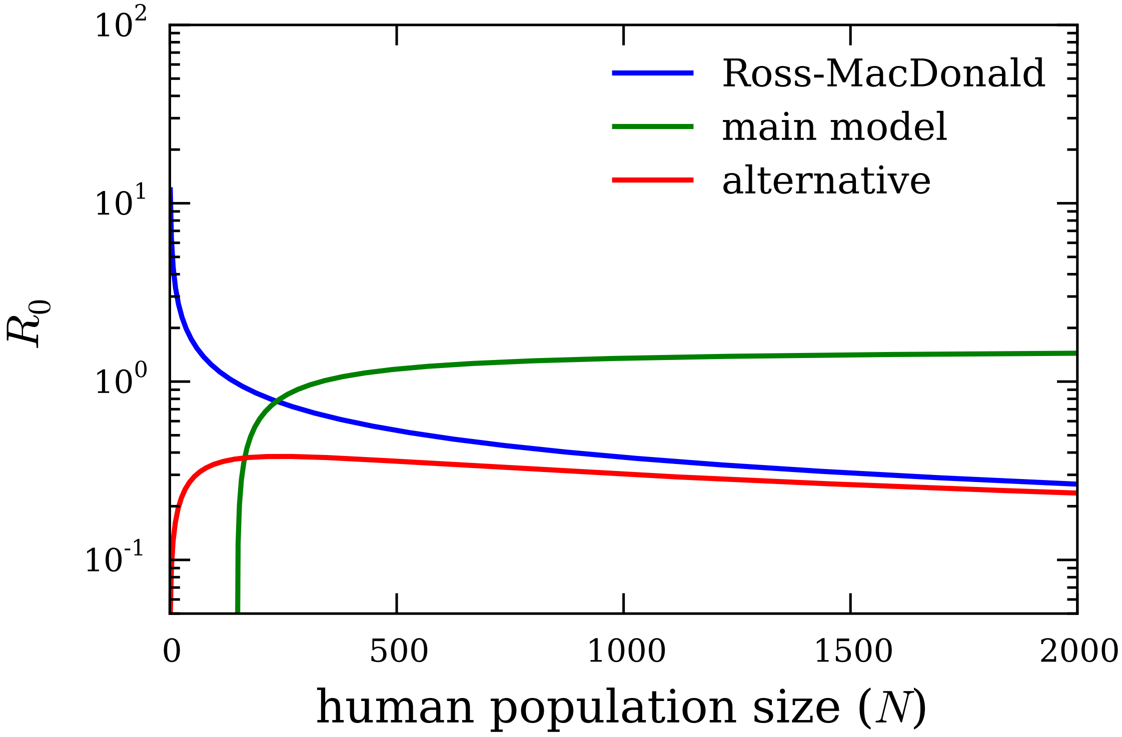

Figure S11. Basic reproduction number (R_0) as a function of the human population size (N), for the three models compared. The other parameter models are the same from Table 1 (main text) for the Marujá.
