# Supplementary material for: Biodiversity Can Help Prevent Malaria Outbreaks in Tropical Forests
Source: PLoS Negl Trop Dis. 2013 Mar 21;7(3):e2139. doi: 10.1371/journal.pntd.0002139 (PMC3605282; doi:10.1371/journal.pntd.0002139)
Supplement: Table S1 — Animal and bird species, density and population size estimates in the Parque Estadual da Ilha do Cardoso. (PDF) [file pntd.0002139.s015.pdf]

Table S1. Animal and bird species, density and population size estimates in the Parque Estadual da Ilha do Cardoso.

Species (popular name)	Animal density	Number of individuals
	(n° of individuals per km^2)	(n°)
Odontophorus capueira (spot-winged wood quail)	26.7 (18.0 - 37.2)	2,963 (2,004 - 4,129)
Ramphastos dicolorus and *R. vitellinus* (toucans)	12.2 (10.6 - 15.9)	1,354 (1,179 - 1,765)
Alouatta guariba (howler monkey)	8.9 (6.9 - 11.6)	997 (769 - 1,291)
Dasyprocta leporina (agouti)	3.4 (2.3 - 4.4)	385 (264 - 488)
Penelope obscura and *P. superciliaris* (guans)	3.2 (2.8 - 4.2)	359 (311 - 467)
Pipile jacutinga (guan)	2.1 (1.8 - 2.7)	234 (203 - 304)
Sciurus ingrami (squirrel)	1.6 (1.1 - 2.0)	182 (124 - 231)
Tinamus solitarius (tinamou)	1.2 (1.0 - 1.6)	140 (121 - 182)

Estimates of animal population size and density were performed in the software DISTANCE 4.1. The values in parentheses correspond to the lower and upper limits of 95% confidence interval. Source: Bernardo [1].

References

1. Bernardo CSS (2004) Abundância, densidade e tamanho populacional de aves e mamíferos cinegéticos no Parque Estadual Ilha do Cardoso, SP, Brasil. Piracicaba: Universidade de São Paulo [Master's thesis]. 156 p.
