# Supplementary material for: Biodiversity Can Help Prevent Malaria Outbreaks in Tropical Forests
Source: PLoS Negl Trop Dis. 2013 Mar 21;7(3):e2139. doi: 10.1371/journal.pntd.0002139 (PMC3605282; doi:10.1371/journal.pntd.0002139)
Supplement: Table S2 — Mosquito species and vegetation types in the Parque Estadual da Ilha do Cardoso. (PDF) [file pntd.0002139.s016.pdf]

Table S2. Mosquito species and vegetation types in the Parque Estadual da Ilha do Cardoso.

Species	Scrub vegetation	Restinga	Tropical pluvial forest
Aedes albifasciatus	13	0	0
Aedes hastatus	3	18	2
Aedes fulvus	0	2	0
Aedes oligopistus	4	26	2
Aedes scapularis	542	79	6
Aedes serratus	26	160	21
Anopheles bellator	26	9	0
Anopheles cruzii	23	33	90
Anopheles homunculus	1	11	15
Anopheles maculipes	1	0	0
Anopheles mediopunctatus	1	0	1
Coquillettidia chrysonotum	766	337	93
Coquillettidia venezuelensis	3	3	1
Culex imitator	1	0	0
Culex inadimirabilis	77	0	0
Culex neglectus	0	2	0
Culex ocossa	2	0	0
Culex ribeirensis	83	0	0
Culex sacchettae	9	15	0
Psorophora albigenu	0	9	0
Psorophora albipes	0	8	0
Psorophora ferox	7	64	0
Limatus durhami	0	42	14
Limatus flavisetosus	0	16	3
Runchomyia cerqueirai	0	5	0
Runchomyia frontosa	0	0	3
Runchomyia humboldti	0	4	1
Runchomyia reversa	0	54	1
Runchomyia theobaldi	0	29	2
Sabethes intermedius	0	1	0
Sabethes soperi	0	1	0
Trichoprosopon pallidiventer	0	2	2
Wyeomyia aporonoma	0	4	3
Wyeomyia confusa	0	11	4
Wyeomyia mulhensi	10	137	8
Wyeomyia occulta	0	1	0
Wyeomyia pallidoventer	0	61	0
Wyeomyia pertinens	0	5	1
Wyeomyia quasilongirostris	12	228	18
Wyeomyia shannoni	0	1	0
Wyeomyia theobaldi	0	17	3
