# Supplementary material for: Biodiversity Can Help Prevent Malaria Outbreaks in Tropical Forests
Source: PLoS Negl Trop Dis. 2013 Mar 21;7(3):e2139. doi: 10.1371/journal.pntd.0002139 (PMC3605282; doi:10.1371/journal.pntd.0002139)
Supplement: Table S3 — Mosquito abundance regression models, independent variables and Akaike Information Criteria values. (PDF) [file pntd.0002139.s017.pdf]

Table S3. Mosquito abundance regression models, independent variables and Akaike Information Criteria values.

Model	Independent variables	*Anopheles cruzii*	*Aedes serratus*	*Runchomyia reversa*	*Limatus durhami*	*Wyeomyia quasilongirostris*
1. GL	Alt	190.89	212.76	165.98	161.03	241.25
2. GL	Bioveg	210.70	209.61	165.72	162.10	239.60
3. GL	Alt + Bioveg	190.83	209.12	162.94	162.19	234.18
4. GL	Alt × Bioveg	192.15	209.10	164.52	156.58	236.10
5. a-LS	Alt	194.81	211.67	173.20	151.96	232.35
6. a-LS	Bioveg	201.69	209.52	169.21	146.64	228.22
7. a-LS	Alt + Bioveg	195.63	210.22	175.46	154.15	229.01
8. a-LS	Alt × Bioveg	205.92	219.95	186.86	165.53	238.09
9. NB	Alt	140.35	183.89	111.82	91.83	195.57
10. NB	Bioveg	149.95	178.85	110.87	83.25	189.81
11. NB	Alt + Bioveg	142.10	177.81	100.49	83.87	181.62
12. NB	Alt × Bioveg	143.95	177.81	86.40	72.64	183.59
13. GA	Alt	139.81	177.94	83.81[a]	62.92	160.22[a]
14. GA	Bioveg	149.86	175.37[a]	86.55	58.41	174.62
15. GA	Alt + Bioveg	136.45[a]	178.23	84.34	57.34[a]	164.58
16. GA	Alt × Bioveg	NC	177.74	NC	NC	NC

Model: GL, Gaussian linear regression model (the simplest model); a-LS, autoregressive least square model (it considered spatial correlation structure); NB, negative binomial regression model (it considered exponential non-linear relationship between mosquito abundance and independent variables); and GA, generalized additive regression model (it considered exponential non-linear relationship and a very flexible smooth function that maximize model fit).
Independent variables: Alt, altitude interpolation; Bioveg, vegetation biomass interpolation; Alt + Bioveg, sum of effects; Alt × Bioveg, interaction.
NC: model did not converge to a result.
[a]: The best model, i.e., the lowest Akaike Information Criteria value.
